# Dynamic *in Situ* Confinement Triggers Ligand-Free Neuropeptide Receptor Signaling

**DOI:** 10.1021/acs.nanolett.2c03506

**Published:** 2022-10-11

**Authors:** M. Florencia Sánchez, Marina S. Dietz, Ulrike Müller, Julian Weghuber, Karl Gatterdam, Ralph Wieneke, Mike Heilemann, Peter Lanzerstorfer, Robert Tampé

**Affiliations:** † Institute of Biochemistry, Biocenter, Goethe University Frankfurt, Max-von-Laue-Str. 9, 60438 Frankfurt am Main, Germany; ‡ Institute of Physical and Theoretical Chemistry, Goethe University Frankfurt, Max-von-Laue-Str. 7, 60438 Frankfurt am Main, Germany; § School of Engineering and Environmental Sciences, University of Applied Sciences Upper Austria, 4600 Wels, Austria; ∥ FFoQSI - Austrian Competence Centre for Feed and Food Quality, Safety & Innovation, FFoQSI GmbH, Technopark 1D, 3430 Tulln, Austria

**Keywords:** G protein-coupled receptors, membrane organization, receptor dynamics, receptor condensates, phase separation

## Abstract

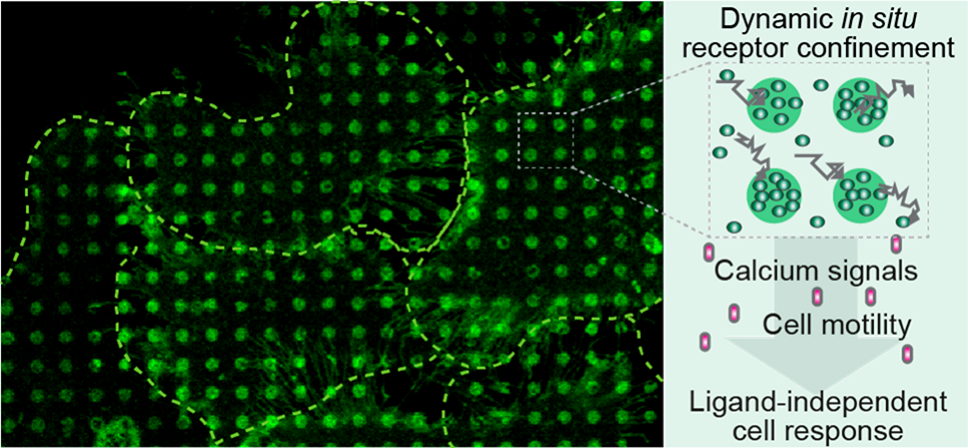

Membrane receptor clustering is fundamental to cell–cell communication; however, the physiological function of receptor clustering in cell signaling remains enigmatic. Here, we developed a dynamic platform to induce cluster formation of neuropeptide Y_2_ hormone receptors (Y_2_R) *in situ* by a chelator nanotool. The multivalent interaction enabled a dynamic exchange of histidine-tagged Y_2_R within the clusters. Fast Y_2_R enrichment in clustered areas triggered ligand-independent signaling as determined by an increase in cytosolic calcium and cell migration. Notably, the calcium and motility response to ligand-induced activation was amplified in preclustered cells, suggesting a key role of receptor clustering in sensitizing the dose response to lower ligand concentrations. Ligand-independent versus ligand-induced signaling differed in the binding of arrestin-3 as a downstream effector, which was recruited to the clusters only in the presence of the ligand. This approach allows *in situ* receptor clustering, raising the possibility to explore different receptor activation modalities.

Cells translate stimuli into biochemical signals through membrane receptors controlling multiple aspects of cell behavior, including migration,^1,2^ differentiation,^3,4^ apoptosis,^5^ as well as infectious diseases and cancer.^6−12^ Receptors form dynamic assemblies or clusters that modulate downstream signaling and final physiological responses. Upon activation, receptors undergo transitions from freely diffusing monomers to less mobile nanoclusters and further to higher-order oligomers, which together with their downstream effectors lead to signaling hubs or dynamic 2D condensates.^13,14^ The mechanism for receptor clustering and its role have become physiologically relevant topics; however, techniques to trigger receptor clustering *in situ* and monitor this assembly process in real time are largely limited.

Nano- and microlithographic approaches have provided cell-compatible scaffolds to investigate confined ligand–receptor interactions. Various techniques, ranging from photolithography^15−17^ to electron-beam lithography^18,19^ and microcontact printing (μCP),^20,21^ have yielded information on how topology and mobility of the stimulus regulate cellular outcomes. One of the main drawbacks of these systems is that the cells are in contact with the functionalized matrices for minutes/hours before the response is evaluated, thus missing the early signaling events upon cluster formation. Recently, optogenetics and optochemistry have enabled the possibility of targeting receptor oligomerization with high spatiotemporal control.^22−24^ However, approaches with minimal perturbance of the system and offering the possibility to analyze large cell populations simultaneously are rare.

Heterotrimeric guanine nucleotide-binding protein (G protein)-coupled receptors (GPCR) are key cell surface proteins that regulate a plethora of cellular responses to external stimuli.^25−27^ The neuropeptide Y_2_ receptor (Y_2_R) belongs to the rhodopsin-like (class A) GPCR superfamily^28,29^ and has been associated with important physiological processes, such as fear extinction and obesity,^30,31^ but also with different cancer types^32−34^ (Supplementary Text 1). Y_2_R activation by neuropeptide Y (NPY) promotes cell migration and proliferation.^35,36^ Spatially restricted cluster formation of Y_2_R was observed *in vivo*; however, the relevance of this confinement remains elusive. Recently, Y_2_Rs responded to light-guided clustering at spatially defined locations.^37^ Receptor activation independently of canonical ligands evoked elevated cytosolic calcium, a change in cell spreading, and a localized migratory pattern.

## Results and Discussion

**Figure 1:**
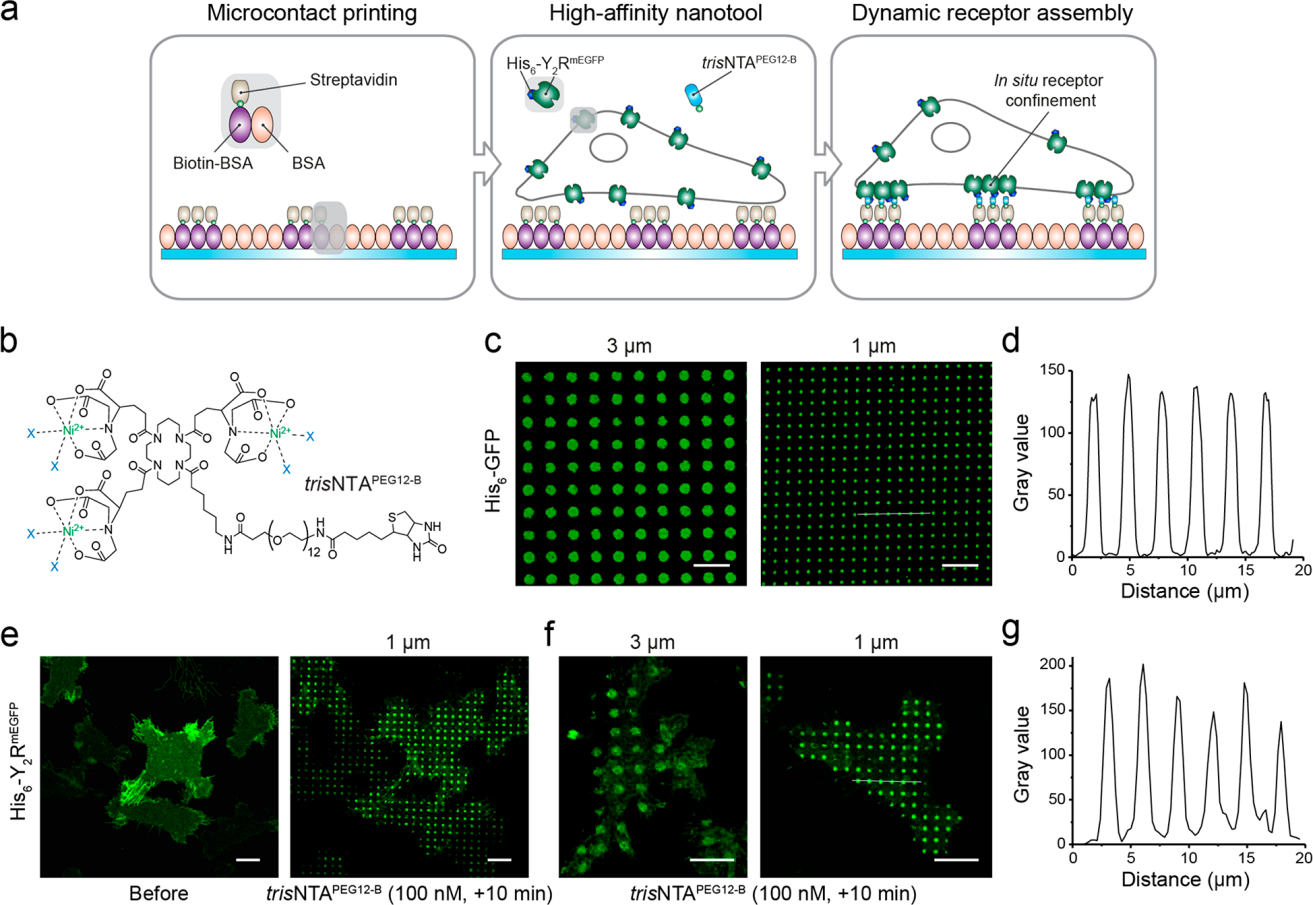
*In situ* ligand-free receptor confinement. (a) Rationale of the experimental design for ligand-free receptor clustering. Matrices prestructured with BSA are stepwise functionalized with biotin-BSA and SA. Upon the addition of the multivalent nanotool *tris*NTA^PEG12-B^, His_6_-tagged receptors in HeLa cells are captured in the prestructured regions via multivalent His-tag/*tris*NTA interaction. (b) Chemical structure of the *tris*NTA^PEG12-B^. (c) Protein patterns of variable size enerated by functionalization of SA matrices with the nanotool followed by incubation with His_6_-GFP (0.1 μM, 20 min). Images were acquired by confocal laser scanning microscopy (CLSM). (d) Intensity profile of the 1 μm pattern (white line in c) reflects high specificity of the interaction. (e) Large-scale cell patterning in living cells occurred 10 min after incubation with the nanotool (*tris*NTA^PEG12-B^ 100 nM final, 10 min). Y_2_R-expressing cells were allowed to adhere to the functionalized matrix for 3 h and immediately imaged by CLSM in live-cell imaging solution (LCIS) at 37 °C. (f) Customized Y_2_R assembly on 3 and 1 μm SA-prestructured matrices. (g) Intensity profile of the 1 μm pattern (white line in f) showed an intensity comparable to that of a soluble His_6_-tagged protein. Scale bars: 10 μm.

We developed a versatile approach to induce dynamic receptor assembly *in situ* based on a multivalent chelator *tris*NTA nanotool (Figure 1a), which is equipped with a biotin moiety (*tris*NTA^PEG12-B^; Figure 1b). This nanometer-sized tool (∼1 nm) displays a high affinity for His_6_-tagged proteins (*K*_D_ ≈ 1–10 nM), resulting in a site-specific and reversible interaction with minimal steric constraints.^38^ Microcontact printing is a widely used method to investigate protein–protein interactions in living cells.^39,40^ However, reproducible patterned substrates with a generic structure over extensive 100 millimeter dimensions, which allow simultaneous analyses of large cell populations, are difficult to produce. We used a large-area perfluoropolyether (PFPE) elastomeric stamp inked with bovine serum albumin (BSA) to print 96-well sized glass.^41,42^ Wells within these plates containing a BSA-microstructured matrix were functionalized with biotinylated BSA (biotin-BSA) and streptavidin (SA; Figure 1a). Subsequent functionalization with the nanotool and His_6_-tagged fluorescent proteins resulted in well-resolved patterns that were analyzed by confocal laser scanning microscopy (CLSM). This result confirmed the specificity of the nickel-loaded *tris*NTA nanotool to capture His_6_-tagged proteins in defined regions of 1 or 3 μm diameter (Figure 1c,d).

To control the organization of membrane receptors, we established a monoclonal human cervical cancer HeLa cell line expressing low amounts of Y_2_R (∼300 000 receptors/cell) utilizing a tetracycline-inducible (T-Rex) expression system.^37^ Y_2_R displayed an N-terminal His_6_-tag to the extracellular space and a cytosolic C-terminal monomeric Enhanced Green Fluorescent Protein (mEGFP; His_6_-Y_2_R^mEGFP^, in brief Y_2_R). These modifications do not affect receptor activity, selectivity, or ligand binding.^37^ It has been demonstrated that Y_2_R does not require the N-terminal region for ligand binding.^43^ Y_2_R-positive cells properly adhered to 1 and 3 μm SA-functionalized matrices and showed a homogeneous receptor distribution at the basal plasma membrane (Figure 1e). The addition of the *tris*NTA^PEG12-B^ nanotool (100 nM final) triggered receptor assembly. Within 5 min, all cells showed receptor clusters at the plasma membrane comparable in size and density (Figure 1e,f, Figure S1). Recruitment of soluble His_6_-tagged GFP proteins as well as Y_2_Rs to 1 μm prestructured spots led to analogous intensity profiles, reflecting that similar densities were obtained in both cases (Figure 1d,g). The specificity of the interaction and the comparison with receptor clustering triggered by an anti-His-tag antibody is provided by additional data sets in the Supporting Information (Supplementary Text 2, Figures S2 and S3).

**Figure 2:**
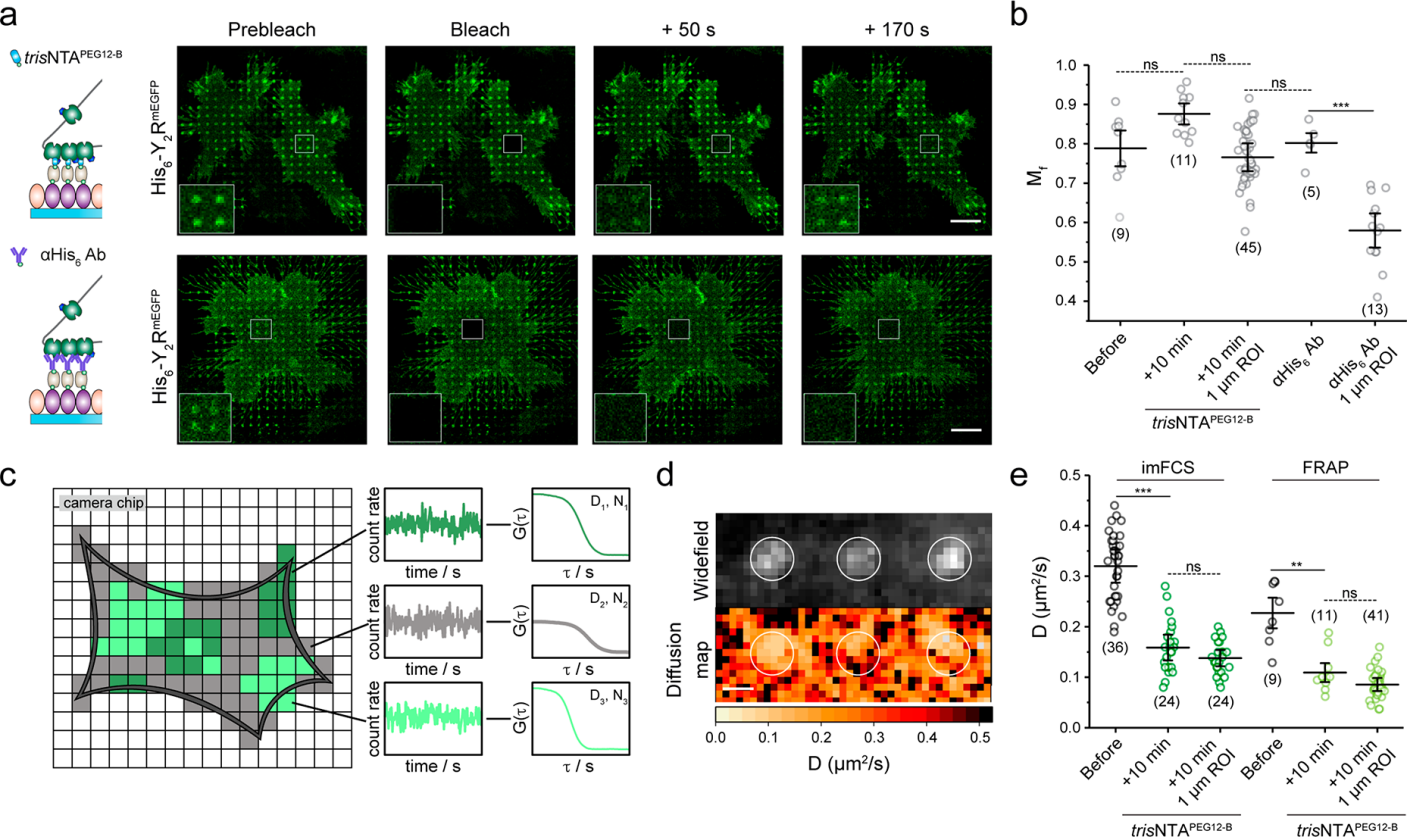
Decrease of receptor mobility in confined regions. (a) FRAP analyses upon Y_2_R clustering induced either by the nanotool *in situ* or by an anti-His_6_ antibody (αHis_6_ Ab). Y_2_R-expressing cells were allowed to adhere to SA- or -αHis_6_ Ab matrices for 3 h and immediately imaged by CLSM in LCIS at 37 °C. The *tris*NTA^PEG12-B^ nanotool was added to a final concentration of 100 nM. Insets represent the bleached ROIs. Fast recovery of the clusters can be detected in the case of the multivalent nanotool. (b) Quantification of the receptor mobile fraction for cell patterning by the *tris*NTA^PEG12-B^ and anti-His_6_ antibody demonstrated an unchanged receptor mobile fraction for the nanotool, suggesting a high receptor exchange. The mean ± SD is shown. Nine cells before, 11 cells after *tris*NTA^PEG12-B^ addition (45 × 1 μm diameter ROIs), and five cells on anti-His_6_ antibody matrices (13 × 1 μm diameter ROIs) were analyzed. ***p ≤ 0.001 for Tukey test. (c) imFCS correlates fluorescence intensity fluctuations in single camera pixels, providing diffusion coefficients with high spatial and temporal resolution. (d) Widefield image of an ROI at the plasma membrane of a living cell upon addition of the nanotool analyzed by imFCS (left). The analyses of numerous pixels simultaneously provide two-dimensional diffusion data that draw a picture of the mobility of membrane receptors and reveal local differences in the diffusion (right). (e) Both techniques demonstrated a decrease in the lateral diffusion of the receptor at the plasma membrane after addition of the chelator nanotool. Analysis of 1 μm clusters within the entire ROI led to a further decrease in the lateral diffusion coefficient. For imFCS analyses, two-sample *t* tests (α = 0.05) were applied to compare the diffusion coefficients for the different conditions. The mean ± SD is shown. 36 and 24 cells for the conditions before and after addition of *tris*NTA^PEG12-B^ were analyzed. For FRAP, the mean ± SD is shown. Nine cells before and 11 cells after *tris*NTA^PEG12-B^ addition (41 × 1 μm diameter ROIs) were analyzed. ***p ≤ 0.001 for Tukey test. Scale bar: 10 μm (a), 1 μm (d).

We examined whether Y_2_R clustering by the chelator nanotool affects lipid diffusion and distribution by labeling the membrane with a lipid-like dye. We observed a homogeneous staining of the plasma membrane, demonstrating that receptor confinement does not affect the lipid distribution (Figure S4). To determine lateral diffusion coefficients (*D*), we performed fluorescence recovery after photobleaching (FRAP). *In situ* receptor clustering was triggered on Y_2_R-expressing cells cultured on SA matrices by incubation with *tris*NTA^PEG12-B^ (100 nM, 10 min), followed by membrane labeling with the lipid-like dye. In a subsequent step, square-shaped regions of interest (ROIs) covering four 1-μm-sized spots were photobleached. Fluorescence recovery was analyzed by a FRAP simulation approach that enabled calculation of *D* independent of bleaching geometry.^44^ The lateral *D* of lipids obtained by FRAP had an average value of *D*_lipid_ = 0.66 ± 0.10 μm^2^/s, which is in agreement with literature values for free Brownian lipid diffusion at the plasma membrane.^45,46^ A significant decrease in the lateral diffusion of the Y_2_R was observed at the basal membrane of cells after receptor confinement by *tris*NTA^PEG12-B^ (*D*_before_ = 0.25 ± 0.08 μm^2^/s versus *D*_after_ = 0.10 ± 0.03 μm^2^/s; Figure 2a,e). Surprisingly, the receptor intensity showed a high recovery within 3 min after photobleaching (Figure 2a, Figure S5, Video 1). Notably, no significant difference in receptor mobile fraction (*M*_f_) before and after addition of the nanotool was observed (*Mf* = 0.80 ± 0.04; Figure 2b). In comparison, FRAP analyses of cells cultured on matrices functionalized with anti-His_6_ antibodies presented a drastic decrease in receptor diffusion and mobile fraction at the clustered spots (*M*_f,anti-His6Ab_ = 0.56 ± 0.08; Figure 2a,b, Video 2). Despite the nanomolar affinity and kinetically stable binding (*k*_off_ = 0.18 h^–1^),^38^ the His-tag/*tris*NTA system relies on molecular multivalency, which enables competition of binding sites with histidine or other histidine-tagged receptors, thus making the process of receptor assembly reversible. We rationalized that free receptors diffuse into the clustered spots and exchange with photobleached receptors at multivalent binding sites, leading to a dynamic confinement. Our results indicate that a high proportion of receptors is exchanged in and out of micrometer-sized clusters, an effect that likely depends on cluster size, with larger clusters showing less recovery.^37^ We also investigated the lateral receptor mobility with a higher spatiotemporal resolution using imaging fluorescence correlation spectroscopy (imFCS; Figure 2c). Multiplexed FCS was realized by analyzing many pixels simultaneously using a widefield setup.^47,48^ ROIs on Y_2_R-expressing cells cultured on SA matrices were analyzed before and after receptor clustering by *tris*NTA^PEG12-B^. Enrichment of Y_2_R at the basal membrane was observed with total internal reflection fluorescence (TIRF) microscopy (Figure 2d). Consistent with the FRAP measurements, the receptor *D* decreased upon cluster formation (*D*_before_ = 0.32 ± 0.06 μm^2^/s and *D*_after_ = 0.16 ± 0.05 μm^2^/s). The receptor diffusion measured before clustering was comparable to membrane proteins of similar size,^49^ demonstrating that the matrix does not affect receptor mobility. ImFCS provides a two-dimensional diffusion map, which resolves local differences in the lateral diffusion coefficient of membrane receptors with high precision. Quantitative analysis of the 1 μm cluster spots in the acquired ROIs resulted in a lateral diffusion coefficient of *D*_spots_ = 0.14 ± 0.03 μm^2^/s (Figure 2e). Taking into consideration that imFCS detects mobile particles only, we determined a similar decrease in lateral diffusion in the patterned regions for cells cultured on matrices functionalized with anti-His_6_ antibodies (Figure S6). We unravel that associations between His_6_-tagged Y_2_Rs and multivalent *tris*NTA^PEG12-B^ resulted in a decreased lateral diffusion but dynamic receptor exchange with an unchanged mobile fraction, which is similar to the behavior described for ligand-activated receptor clustering.^50^

**Figure 3:**
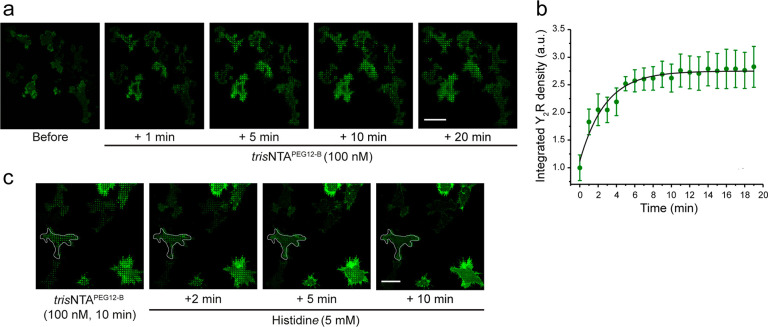
*In situ* receptor clustering with high spatiotemporal resolution. (a) Time-lapse imaging of Y_2_R assembly. Y_2_R-expressing HeLa cells were allowed to adhere to prestructured SA matrices for 3 h and were visualized by CLSM in LCIS at 37 °C. Time-lapse images were recorded for 20 min immediately after the addition of *tris*NTA^PEG12-B^ (100 nM). Scale bar: 20 μm. (b) Receptor-integrated density in the patterned regions increased monoexponentially, leading to an assembly rate of 0.35 ± 0.05 min^–1^ and τ_1/2_ = 3 min (50–200 × 1 μm ROIs per experiment were analyzed from a total of 30 cells from three independent experiments, 10 cells per experiment). (c) Reversal of the interaction and disassembly of the clusters is demonstrated upon the addition of histidine. Y_2_R-expressing cells were allowed to adhere to the SA matrices for 3 h, and then receptor confinement was induced by the addition of *tris*NTA^PEG12-B^ (100 nM). Subsequently, cells were incubated with histidine (5 mM) for 2 to 10 min followed by washing. Scale bar: 10 μm.

To induce receptor clustering with high spatiotemporal resolution, we cultured cells over 1 μm matrices and tracked the receptor redistribution by CLSM after *in situ* addition of the multivalent nanotool. Receptor clustering occurred in the first minutes and increased within 10 min until an equilibrium was reached, resulting in a 2.5-fold increase in receptor density in the clustered regions compared to the initial state (Figure 3a, Video 3). The kinetic profile of Y_2_R recruitment to the 1 μm spots followed a pseudo-first-order assembly rate of 0.35 ± 0.05 min^–1^ (Figure 3a,b). Considering the average cell area of 1420 ± 50 μm^2^ and the enrichment factor (2.5-fold), we estimated a receptor density of ∼400 receptors per 1 μm circular spot, which is comparable to other receptor clusters.^51,52^ The addition of histidine to patterned cells resulted in rapid and complete disassembly of the receptor clusters, demonstrating the reversibility of the system, a key advantage to investigate receptor dynamics (Figure 3c). Overall, this approach presents the advantage of monitoring cluster formation in real-time, compared to established systems using matrices with immobilized ligands or antibodies, and requires lower concentrations. The nanotool can also be adapted to a variety of systems and receptors through minimal modifications.

**Figure 4:**
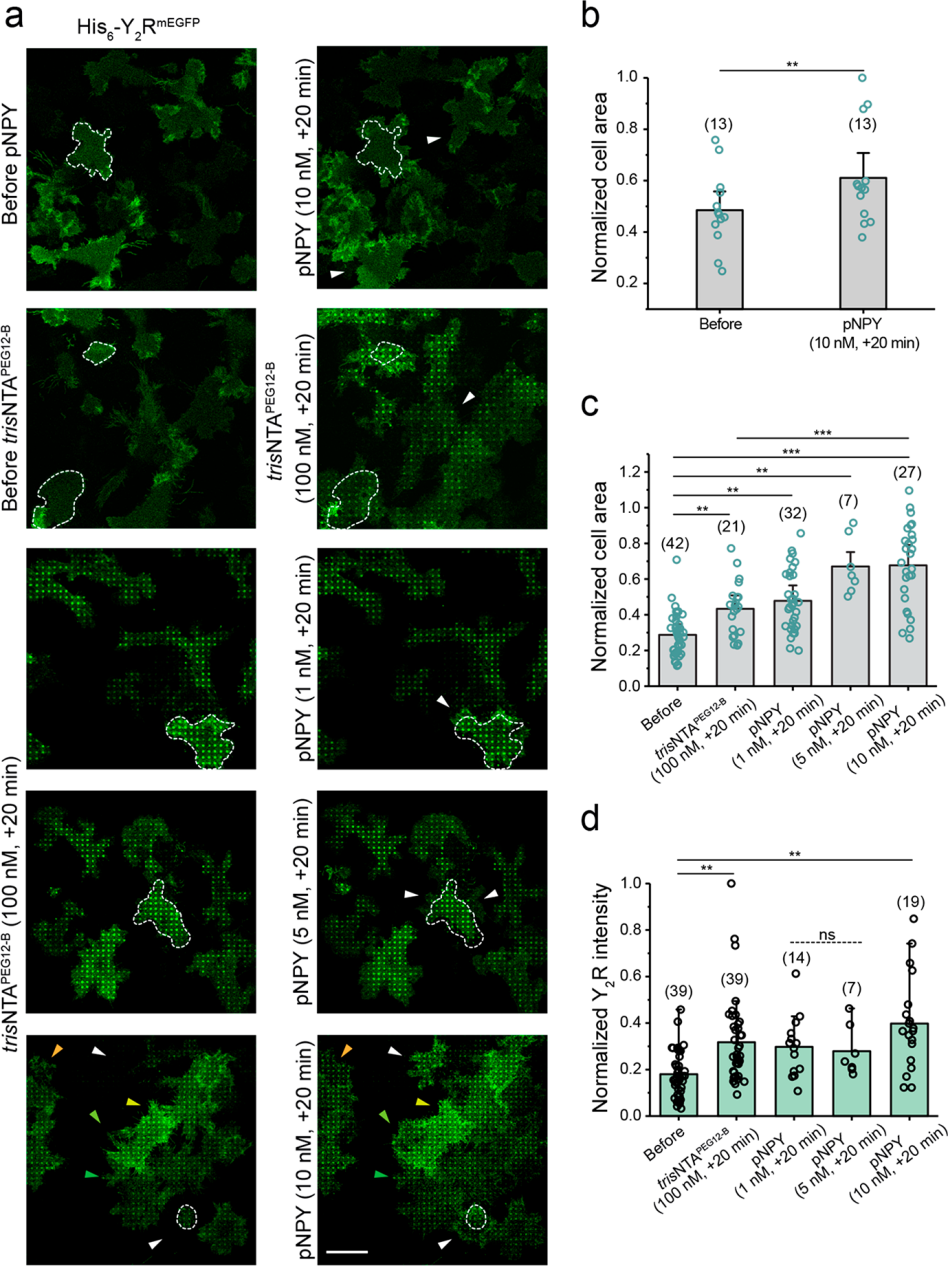
Receptor clustering amplifies the cell response induced by ligand activation. (a) Confocal microscopy images of cells expressing Y_2_R exposed to different conditions. Y_2_R-expressing HeLa cells were allowed to adhere to prestructured SA matrices for 3 h and visualized by CLSM in LCIS at 37 °C. Cells were visualized and imaged for 20 min after the addition of *tris*NTA^PEG12-B^ or pNPY or both, first *tris*NTA^PEG12-B^ and subsequently pNPY (20 min incubation time, each). Scale bar: 20 μm. (b) Cell area analysis before and 20 min after the addition of pNPY (10 nM) showed a 20% area increase, confirming an effect of ligand activation on cell motility. Values for cell area were normalized with respect to the highest value. The mean ± SD (13 cells) is shown. **p ≤ 0.01 for Tukey test. (c) Cell area analysis before and 20 min after the addition of *tris*NTA^PEG12-B^ (100 nM) and subsequent addition of pNPY (1, 5, and 10 nM, one well for each concentration) showed a dose-dependent area increase, demonstrating an amplification effect of receptor clustering in combination with pNPY. Cell area values were normalized with respect to the highest value. The mean ± SD (42 cells before, 21 cells after *tris*NTA^PEG12-B^ and 14, 7, 19 for pNPY 1, 5, and 10 nM, respectively) is shown. **p ≤ 0.01 and ***p ≤ 0.001 for the Tukey test. (d) Quantification of receptor intensity in the nanotool-induced patterned regions showed a significant increase in pattern intensity after the addition of pNPY (10 nM), the concentration that had the largest effect on cell motility. The mean ± SD is shown (19–39 cells and 50–220 × 1 μm ROI, were analyzed). ***p ≤ 0.001 for the Tukey test.

We next evaluated the physiological relevance of Y_2_R clustering. Y_2_R activation by its natural ligand NPY promotes cell migration and proliferation.^35,36^ In cells cultured on SA matrices, a 17% increase in the cell area was detected after addition of the agonist porcine neuropeptide Y (pNPY, *K*_D_ = 5.2 ± 2.0 nM; Figure 4a,b). When clustering was induced by the nanotool, we also observed a fast change in cell spreading and motility and a 20% increase in the total cell area concomitant to receptor assembly (Figure 4a,c). This analogous effect indicates a ligand-independent response to receptor clustering. We did not observe change in cell motility upon addition of the *tris*NTA^PEG12-B^ in cells cultured on matrices without SA (Figure S7). Cells expressing Y_2_R^mEGFP^ (lacking a His_6_-tag) on SA matrices showed no significant change in cell spreading upon addition of the nanotool, demonstrating the specificity of the response (Figure S7). Next, we determined the increase in cell area upon ligand-induced activation in cells that were non- and preclustered by the nanotool. Strikingly, receptor clustering by the nanotool amplified the motility effect induced by the pNPY. In preclustered cells, stimulation with pNPY (10 nM) led to a 2-fold amplification and a 40% increase in cell area compared to the initial state. A dose-dependent increase in cell area (Figure 4a,c) and cluster intensity (Figure 4a,d) was observed for *tris*NTA^PEG12-B^-preclustered cells. Overall, these results indicate a critical function of the receptor clusters, an amplification of the signal in prepatterned cells, or, from the other point of view, a sensitization of the receptor to lower concentrations of the natural ligand.

As calcium signals are known to regulate cell motility, we monitored local calcium dynamics utilizing a far-red cell-permeable calcium-sensitive dye. By dual-color imaging, receptor assembly and the cytosolic calcium concentration were simultaneously recorded in living cells over the matrices. Upon addition of *tris*NTA^PEG12-B^, receptor recruitment led to a 2-fold increase in cytosolic calcium concentration with a rapid rise within 2 min (Figure S8). A second increase in Ca^2+^ signals was detected upon subsequent addition of pNPY (10 nM final). To confirm the specificity of the Ca^2+^ response, cells were cultured on control matrices without SA. No calcium signal was observed in these cells (Figure S9). To demonstrate an enhancement of the calcium response induced by the ligand, we monitored calcium signals in non- or nanotool-preclustered cells (Figure S10). In preclustered cells, we observed a 1.6-fold increase in cytosolic calcium signal upon pNPY stimulation compared to the initial state. In contrast, in nonclustered cells, a 1.2-fold increase was detected upon addition of pNPY.

Our results showed analogous calcium signaling for ligand-free versus ligand-induced systems and an amplification of the signal for ligand-induced activation in preclustered cells. Y_2_R has been found in a conformational equilibrium between inactive and active states in the absence of the ligand and forms high-affinity active complexes with G_αi_ proteins.^53^ By ligand-free receptor clustering, the high local receptor density may increase the residence time of G proteins in vicinity and recruit further downstream effectors, which could boost the probability of activation and subsequent signaling. Based on the formation of the high affinity Y_2_R/G_αi_ protein complexes and the short time regime (1–5 min) in which changes in Ca^2+^ concentration and cell motility are observed, it is likely that the ligand-independent activation mechanism involves the G protein pathway. G protein signaling leads to the release of Gβγ and activation of phospholipase Cβ, which cleaves phosphatidylinositol 4,5-bisphosphate into diacylglycerol and phosphatidylinositol (3,4,5)-trisphosphate (PIP_3_). PIP_3_ opens intracellular calcium stores through PIP_3_ receptors, leading to local activation of cytoskeletal proteins and the observed cell motility response. We observed actin reorganization and an actin enrichment at the cell periphery upon addition of the nanotool, suggesting that clustering enhances signaling to actin polymerization (Figure S11). Unraveling the regulatory proteins that modulate the actin rearrangement will require the use of a combination of techniques such as TIRF microscopy and single-molecule tracking experiments.

**Figure 5:**
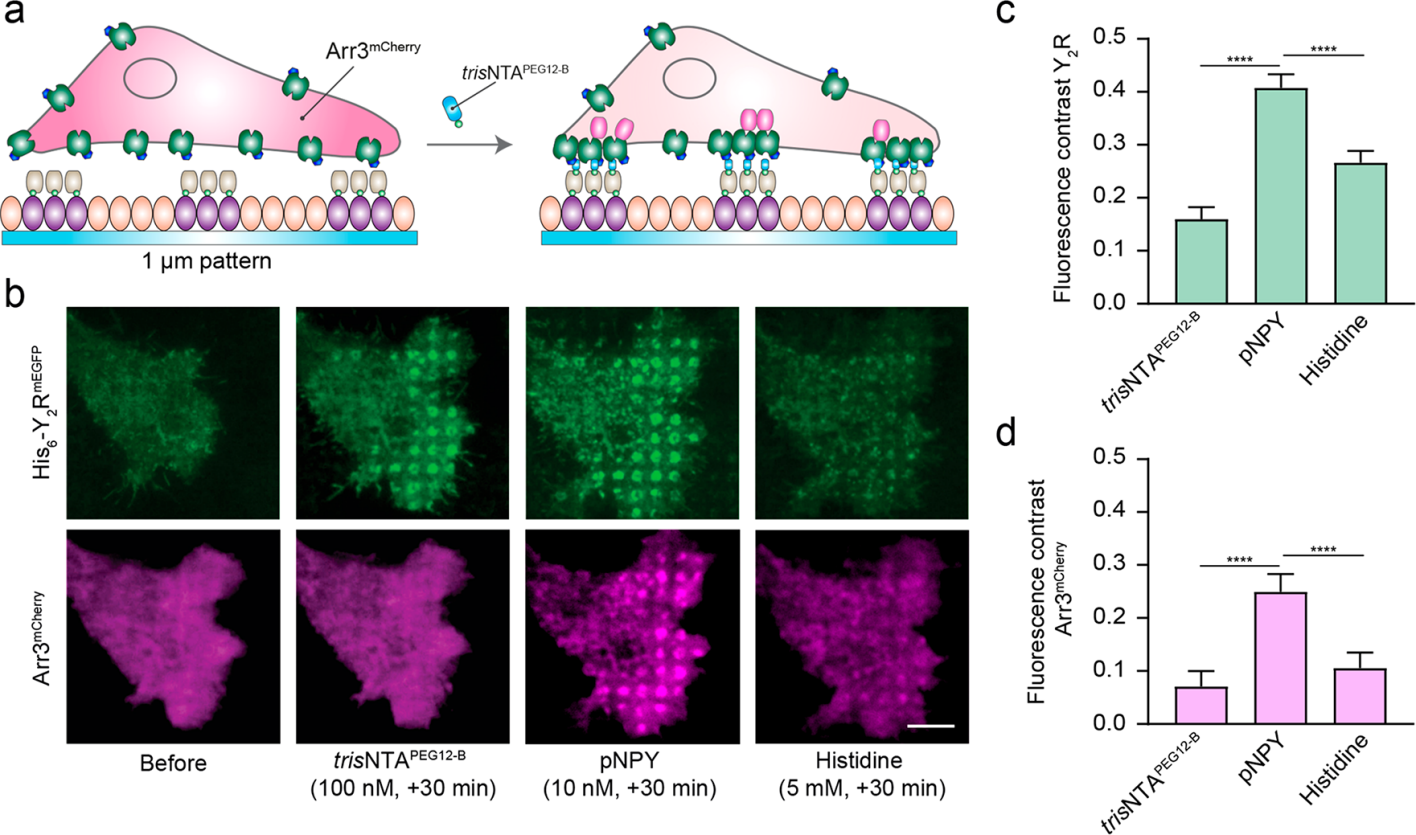
Arrestin-3 recruitment upon ligand-induced receptor activation. (a) Schematic representation of the experimental setup. Cells coexpressing Y_2_R and Arr3 were allowed to adhere to SA-prestructured matrices for 3 h and visualized by TIRF microscopy in LCIS at 37 °C. (b) Representative TIRF images of cells before and upon addition of *tris*NTA^PEG12-B^ (100 nM, 30 min) and subsequent incubation with pNPY (10 nM) and histidine (5 mM) in LCIS for 30 min at 37 °C. All concentrations mentioned are final concentrations in the wells. Scale bar: 5 μm. (c) Quantification of the fluorescence contrast in the patterned regions for Y_2_R confirmed receptor enrichment upon the addition of *tris*NTA^PEG12-B^ (2-fold with respect to the basal signal before, 100 nM, 30 min), which further increased 4-fold upon the addition of pNPY (10 nM, 30 min). Histidine addition led to a decrease in the signal (1.7-fold decrease compared to pNPY, 5 mM, 30 min). Data were normalized with respect to the fluorescence intensity before clustering and are displayed as the means ± SEM (60 cells for each condition were analyzed). Tukey’s multiple comparison test was applied (***p ≤ 0.001). (d) Fluorescence contrast analysis demonstrated no significant recruitment of Arr3 upon *tris*NTA^PEG12-B^ (1.4-fold with respect to the basal signal before, 100 nM, 30 min). Addition of pNPY increased the Arr3 signal (3.6-fold, 10 nM, 30 min), confirming copatterning of the downstream signaling molecules. Subsequent addition of histidine led to a decrease in the signal (2.3-fold, 5 mM, 30 min). Data were normalized with respect to the fluorescence intensity before clustering, and it is expressed as the means ± SEM (60 cells for each condition were analyzed). Tukey’s multiple comparison test was applied (***p ≤ 0.001).

We finally explored the impact of receptor clustering on downstream signaling by monitoring arrestin-3 (Arr3) recruitment. GPCR desensitization involves a complex series of events, e.g., receptor phosphorylation, arrestin-mediated internalization, receptor recycling, and lysosomal degradation.^53^ Short-term desensitization occurs within minutes and is primarily associated with arrestin preventing G protein interaction with the GPCR. Arrestins bind to activated, phosphorylated GPCRs and block receptor-G protein interaction by steric hindrance at the receptor-coupling interface, while serving as adaptors for key components of the endocytic machinery and numerous signaling proteins.^25,54^ In the presence of high concentrations of the canonical ligand, an Arr3-dependent internalization, subsequent endosomal sorting, and recycling of Y_2_R to the cell membrane were observed.^55,56^ However, recent studies demonstrated a strong and persistent activation of the G_αi_ pathway upon Y_2_R activation, which may deplete the intracellular G protein repertoire before Arr3 binding can terminate signaling.^53^ To assess whether ligand-free clustering leads to Arr3 recruitment, we transfected cells stably expressing the Y_2_R with Arr3^mCherry^ (in brief Arr3) and monitored Arr3 recruitment in real-time by TIRF microscopy (Figure 5a).

In agreement with our results shown above, image analysis at an equilibrium state (30 min after addition of the nanotool) showed a subsequent increase in Y_2_R density in the clustered regions upon addition of pNPY (Figure 5b,c). Surprisingly, upon receptor confinement by the nanotool, we did not observe a significant increase in Arr3 recruitment by intensity-contrast analysis of the patterned spots, whereas a significant Arr3 recruitment was detected upon addition of pNPY (Figure 5b,d). Reversibility by specific competition with histidine showed that half of the intensity in the patterned regions was dissipated of the Y_2_R/Arr3 assemblies (Figure 5b,d). These results suggest that not all receptors within the cluster regions are associated with the nanotool upon addition of the ligand, supporting the observation of increased receptor density in the presence of the pNPY. Patterning of Arr3 was also detected in cells on an anti-His_6_ antibody matrix within the first minutes after addition of pNPY (Figure S12). In this case, we did not observe a significant change in receptor density upon addition of the pNPY, indicating that the high degree of immobilization and large size of the antibody might restrict the transient enrichment of active receptors into the clustered regions.

Specific clusters termed GPCR hot spots (40–300 nm) have been visualized at the plasma membrane of living cells.^25,50,57,58^ These hot spots represent regions that preferentially engage signaling and that are enriched in both receptors and G proteins. We hypothesize that the induced microscale clusters trigger the formation of hot spots, which provide an ideal environment for recruitment of more active receptors and thus amplification of the signal. By increasing the local effective receptor concentration, this organization may amplify both the speed and efficiency of receptor-G protein coupling while enabling local signal transduction. Our results show a difference between Arr3 recruitment in the ligand-free mode compared to the ligand-activated state. These observations indirectly confirm a high-affinity interaction between the Y_2_R and Gα_i_ and suggest active recruitment of G proteins.^53^ Likewise, the increased recruitment of receptors observed after addition of pNPY may be directly related to the dynamic nature of the confined regions.

In summary, we unraveled a ligand-independent receptor activation upon clustering and an amplification of the motility and calcium signals upon ligand-induced activation in cells preclustered with the nanotool. These new findings underline the importance of investigating the basic signaling pathways behind Y_2_ receptor activation and its migratory response. The NPY ligand plays an important role in the nervous, immune, and endocrine systems^59−61^ and can affect the proliferation, apoptosis, differentiation, and migration of different cell types.^62^ In addition, NPY has been found to play a role in the progression of diverse types of cancer and diseases such as brain, bone, and breast cancer, as well as osteoporosis.^32,33^ NPY or analogous small peptide agonists were tested as potential new strategies for the diagnosis or treatment of breast cancer and osteoporosis.^33^

Regarding receptor clustering, spatially restricted ligand–receptor interactions were observed *in vivo*. In neurons, Y_2_ is highly expressed, and the receptors are not evenly distributed across the cell.^63^ Hence, a local receptor network of prestabilized ligand–receptor complexes may be critical for homeostasis, modulation, and plasticity of cortical circuits.^64^ Our nanotool approach is physiologically relevant because receptor clustering and cell responses can be followed in real-time. Most pharmacological studies on Y_2_R signaling involve the use of high ligand concentrations, which in many cases are not physiologically relevant. The ligand-independent response we observed may help to change the perception of NPY-induced signaling and highlights the relevance of receptor clustering. Understanding how Y_2_R clustering modulates calcium signals and cell migration may be crucial for the future development of cancer therapeutics involving NPY or synthetic agonists. For example, engineered preoligomers of ligand or agonist peptides may have a more potent effect than higher concentrations of soluble monomeric NPYs.

## Supporting Information Available

Experimental materials and methods, supplementary text and references, additional experimental results including controls, receptor diffusion analysis on antibody-functionalized matrices, lipids diffusion analysis, calcium measurements under all of the conditions (PDF)
Video 1: Time-lapse after photobleaching of a region of interest containing Y2R clusters induced by the nanotool. A fast recovery is observed within the first minutes indicating a high dynamic behavior for these clusters. (AVI)
Video 2: Time-lapse after photobleaching of a region of interest containing Y2R clusters induced by an anti-His6 tag antibody immobilized on the pre-structured matrices. There is no recovery of the clusters indicating a high degree of immobilization (AVI)
Video 3: Time-lapse immediately after the addition of the nanotool to Y2R expressing cells over SA pre-structured matrices show fast enrichment of the receptors in real-time(AVI)

## Supplementary Material

nl2c03506_si_001.pdf

nl2c03506_si_002.avi

nl2c03506_si_003.avi

nl2c03506_si_004.avi
