# Supplementary material for: Dynamic in Situ Confinement Triggers Ligand-Free Neuropeptide Receptor Signaling
Source: Nano Lett. 2022 Oct 11;22(20):8363–71. doi: 10.1021/acs.nanolett.2c03506 (PMC9614963; doi:10.1021/acs.nanolett.2c03506)
Supplement: Supplementary file 1 — nl2c03506_si_001.pdf [file nl2c03506_si_001.pdf]

Dynamic *in situ* confinement triggers ligand-free neuropeptide receptor signaling

M. Florencia Sánchez[1], Marina S. Dietz[2], Ulrike Müller[3], Julian Weghuber[3,4],

Karl Gatterdam[1], Ralph Wieneke[1], Mike Heilemann[2], Peter Lanzerstorfer[3], Robert Tampé[1,*]

[1]Institute of Biochemistry, Biocenter, Goethe University Frankfurt, Max-von-Laue-Str. 9, 60438 Frankfurt am Main, Germany; [2]Institute of Physical and Theoretical Chemistry, Goethe University Frankfurt, Max-von-Laue-Str. 7, 60438 Frankfurt am Main, Germany; [3]School of Engineering and Environmental Sciences, University of Applied Sciences Upper Austria, Wels, Austria; [4]FFoQSI - Austrian Competence Centre for Feed and Food Quality, Safety & Innovation, FFoQSI GmbH, Technopark 1D, 3430 Tulln, Austria

*To whom correspondence may be addressed Email: tampe@em.uni-frankfurt.de

Material and Methods	page 2-10
Supplementary Text 1	page 11
Supplementary Text 2	page 12
Supplementary References	page 13
Supplementary Figure S1-S12	page 14-26
Supplementary Video 1-3	page 27

SUPPORTING INFORMATION

MATERIALS AND METHODS

***Synthesis of trisNTA$^{PEG12-B}$*.** Cyclam-Lys-*tris*NTA[1] (5.0 mg, 4.8 µmol), Biotin-PEG$_{12}$-NHS (23.0 mg, 24.0 µmol) and DIPEA (12.2 µL, 72.0 µmol) were dissolved in 0.5 ml dry DMF and stirred for 2 h at RT. After reaction, the volatile components were removed by lyophilization. Raw product was purified by reverse-phase (RP)-HPLC (mobile phase A: H_2O + 0.1% TFA, B: acetonitrile + 0.1% TFA; gradient 5% to 80% B in 20 min; MZ-PerfectSil, 300 ODS, 5 µm, 250 x 10 mm, flow 4 ml/min). A biotin moiety was integrated into the nanotool for immobilization to SA in the pre-structured matrices. The PEG$_{12}$ linker between the biotin and the *tris*NTA unit increased the flexibility of the molecule. The identity of *tris*NTA$^{PEG12-B}$ was confirmed by liquid chromatography-coupled mass spectrometry (LC-MS, Waters BioAccord System). Datasets were recorded with an ACQUITY UPLC I-Class Plus chromatography system and ACQUITY RDa Detector, which was set to a cone voltage of 25 V, capillary voltage of 1.2 kV and a desolvation temperature of 500 °C operating in positive ionization mode. For reverse-phase separation, an ACQUITY UPLC Peptide BEH C18 column (300 Å, 1.7 µm, 2.1 mm x 100 mm) was used (Figure S13). *tris*NTA$^{PEG12-B}$ was dissolved in HBS buffer (20 mM HEPES-NaOH pH 7.5, 150 mM NaCl) and incubated with 10-fold excess of $NiCl_2$. After 30 min incubation at 4 °C, the excess of Ni(II) was separated by a size exclusion chromatography gravity column (PD MidiTrap G-10). The synthetized compound presents no toxicity and high stability under a broad range of temperatures and pH values and is soluble in water as well as buffer solutions such as PBS, HBS or tris buffer.

***Microcontact printing*.** Large-area microcontact printing was performed as described previously[2]. In brief, a field of a large-area PFPE elastomeric stamp (1 µm grid size), obtained by the EV-Group (St. Florian am Inn, Upper Austria, Austria), was cut out, and washed by flushing with ethanol (100%) and distilled water. After drying with nitrogen, the stamp was incubated in 50 ml BSA solution (1 mg/ml, Sigma-Aldrich) for 30 min followed by washing the stamp with phosphate-buffered saline (PBS) and distilled water. After drying with nitrogen, the stamp was placed with

homogeneous pressure onto a clean epoxy-coated glass substrate (Schott Nexterion Slide E) and incubated overnight at 4 °C. The next day, the stamp was stripped from the glass with a forceps, and the microstructured glass was bonded to a 96-well plastic casting using an adhesive tape (3M) and closed with an appropriate lid.

Functionalization of the pre-structured matrices. BSA-pre-structured wells were incubated with biotin-BSA (0.1 mg/ml, Thermo Fisher Scientific, Waltham, MA, USA) and SA (1 µM, Sigma-Aldrich, Munich, Germany) in PBS, each for 1 h at RT. Incubated wells were thoroughly washed with PBS after each step to remove unbound biotin-BSA and SA. For binding of soluble His-tagged proteins, wells were incubated with *tris*$NTA^{PEG12\text{-}B}$ (0.5 µM) in HBS buffer for 1 h at RT. For nickel(II) loading, the pre-structured matrices were sequentially incubated with imidazole (1 M, 2 min), EDTA (100 mM, 2 min), and $NiCl_2$ (10 mM, 5 min). Wells were carefully washed after each step. Finally, HBS buffer containing EDTA (50 µM) was used to remove the free, non-complexed nickel ions. His_6-GFP (100 nM) previously expressed and purified was added to the wells and incubated for 30 min at RT. Experiments were performed in biological replicas (N=5).

Cell culture. HeLa Flp-In^{TM} T-Rex^{TM} Y_2R cells (His_6-Y_2R^{mEGFP} or Y_2R^{mEGFP}) were generated and cultured at 37 °C, 5% CO_2, and 95% humidity[3]. For culturing the stable cell line, high glucose Dulbecco's modified Eagle's medium (DMEM) (Gibco/Thermo Fisher Scientific) was supplemented with 10% tetracycline-free fetal calf serum (FCS, Bio&Sell), blasticidin S HCl (1 µg/ml, Thermo Fisher Scientific), and hygromycin B (50 µg/ml, Thermo Fisher Scientific). To induce receptor expression the cell medium was replaced with fresh medium containing tetracycline (0.1 µg/ml, Fluka) 18 h before imaging. The same concentration of tetracycline resulting in an efficient plasma membrane targeting was used for all the experiments. The cells were regularly tested for mycoplasma contamination.

***Receptor confinement in real-time by trisNTA$^{PEG12\text{-}B}$*.** Cells expressing Y_2R (His_6-Y_2R^{mEGFP} or Y_2R^{mEGFP}) were trypsinized and allowed to adhere to SA pre-structured matrices for 3 h or overnight. 15-18 h prior to the experiment, the cell medium was replaced with fresh medium containing tetracycline (0.1 µg/ml) to induce receptor expression. The cells were visualized by CLSM in live-cell imaging solution (LCIS, Thermo Fisher Scientific) at 37 °C. Cells were subsequently incubated with nickel-loaded *tris*NTA$^{PEG12\text{-}B}$ (final concentration 100 nM) in LCIS for 10-15 min at 37 °C. For reversibility experiments, micropatterned cells were incubated with histidine (5 mM) in LCIS for 2 to 10 min followed by washing with LCIS. To avoid photo-bleaching in our recordings, we recorded an image every minute after adding the nanotool. We applied control of the focus by the 'definite focus' tool, which allowed to always image the same z-position by re-focusing before taking every image. We generated a monoclonal HeLa cell line, which was established for low expression of the Y_2 receptor. This led to a more homogeneous cell population with respect to receptor expression. Slight cell-to-cell variations were still observed. Experiments were performed in biological replicas (N=4).

***Receptor confinement on antibody-micropatterned matrices*.** Wells pre-structured with BSA were subsequently incubated with biotin-BSA (0.1 mg/ml), SA (1 µM), and a biotinylated anti-His_6 antibody (1 µM) (ab106261, Abcam) in PBS for 1 h at RT. Wells were washed thoroughly with PBS to remove unbound antibody. Cells expressing Y_2R were trypsinized and seeded onto the antibody patterns. After 3 h, cells were visualized by CLSM in LCIS at 37 °C. Experiments were performed in biological replicas (N=5).

***Time-lapse calcium imaging*.** 18 h after seeding the cells onto pre-structured SA-matrices, cells were incubated with BioTracker 609 Red Ca^{2+} AM dye (3 µM, Merck Millipore) in fresh medium for 30 min. The cell-membrane permeable dye is de-esterified by cellular esterases and remains trapped in the cytosol. After incubation with the Ca^{2+} dye, cells were rinsed three times with PBS and imaged by CLSM in LCIS at 37 °C. For investigation of Ca^{2+} signal, time-lapse images were

taken (5 slices z-stacks, 45-s interval) before and after addition of *tris*$NTA^{PEG12-B}$. Fluorescence intensity ($\lambda_{ex/em}$ 590/609 nm) of the dye changes depending on the intracellular Ca^{2+} concentration. Maximum intensity projections of single channels were analyzed. The ImageJ ROI tool was used to define the areas of the image to be analyzed. We consider a ROI covering the complete cell contour. Mean gray values (F) were background subtracted and normalized to the fluorescence in cells before F_0. Experiments were performed in biological replicas (N=3).

Plasma membrane staining. Live-cell membrane staining was performed directly after receptor assembly in living cells grown on pre-structured matrices. CellMask™ deep red plasma membrane stain (Thermo Fisher Scientific) was used according to manufacturer's instruction. 1 µl of the stock solution (1000x dilution) was dissolved in 1 ml of warm LCIS (final concentration 5 µg/ml) and subsequently added to the cells, incubated for 5 min at 37 °C, and washed with LCIS before visualization. Experiments were performed in biological replicas (N=3).

F-actin staining. Live-cell F-actin staining was performed in the microscope in living cells grown on pre-structured matrices. SiR-actin (Cytoskeleton Inc) was used according to manufacturer's instruction. 1 µl of the stock solution (1 mM in DMSO) was dissolved in 1 ml of warm LCIS (final concentration 1 µM) and subsequently diluted to be added to the cells to a final concentration of 100 nM. The SiR-actin dye was incubated for 15 min, washed with LCIS and cells were visualized at 37 °C in LCIS. Cells were imaged before, during and after nanotool-induced receptor clustering. Experiments were performed in biological replicas (N=3).

Confocal laser scanning microscopy. Images were recorded by using a CLSM Zeiss LSM 880 (Carl Zeiss) equipped with a Plan-Apochromat 63x/1.4 Oil DIC M-27 objective. Sequential settings for dual-color imaging were used. Excitation wavelengths for the different fluorophores: 488 nm (argon laser) for mEGFP; 594 nm for the Ca^{2+} dye; 633 nm (helium-neon laser) for the plasma membrane dye. Signals were detected after appropriate filtering on a photomultiplier. Intensities

of channels were adjusted over the whole image for better visualization of overlap and exported by Zen blue (version 2.3 lite, Zeiss). Detector amplification, laser power, and pinhole were kept constant for all studies.

Image analysis. Fluorescence images were processed with Zen blue, ImageJ, and Fiji software.[4,5] All images were background subtracted. Integrated density, mean gray value and cell area were obtained with ImageJ. Data were plotted with OriginPro.

Fluorescence recovery after photobleaching. FRAP experiments were conducted at the CLSM Zeiss LSM 880 using 63 x/1.4 Oil DIC objective. Rectangular-shaped regions (6-10 μm radius) were bleached within 10 s with high laser intensities. Fluorescence recovery was monitored by repetitively imaging an area containing the photobleached region at 0.1 frame/s for ∼150 s. For the analysis, a simulation approach that allows computation of diffusion coefficients regardless of bleaching geometry used in the FRAP series was applied.[6] The method is based on fitting a computer-simulated recovery to actual recovery data of a FRAP series. The algorithm accepts a multiple-frame TIFF file, representing the experiment as input, and simulates the diffusion of the fluorescent probes (*2D* random walk) starting with the first post-bleach frame of the actual data. Once the simulated recovery is finished, the algorithm fits the simulated data to the real one and extracts the diffusion coefficient. The algorithm iteratively creates a series of simulated images, where each frame corresponds to a single iteration. The intensity values are extracted from the (user indicated) bleached area of the simulated frames, thus determining the general shape of the recovery curve. The "time" axis at this stage is in arbitrary units (iterations). To extract the diffusion coefficient, the simulated recovery curve needs to be fitted to the real recovery curve, by appropriately stretching the "time" axis. The time between frames in the actual data set is obviously known, thus once overlapping optimally the simulated curve with the real one, the duration of one iteration, in real-time units, is determined. The diffusion coefficient of the simulated series is then calculated according to eq. 1, where D_S is the simulation-extracted diffusion

coefficient, l is the step of a molecule in each iteration of the simulation, corresponding to one pixel in the image (the pixel size is calibrated previously, by imaging a known calibration sample), and t_i is the time interval between steps (determined as explained).

$$D_s = \frac{l^2}{4t_i} \quad (1)$$

The simulation proceeds until a plateau is reached (equilibration of the fluorescence intensity in the bleached area). The number of data points in the simulated recovery is typically different (larger) than the number of experimental points. In addition, the real experimental data may not have been acquired until equilibration of fluorescence. To determine t_i, the algorithm scans a range of possible values for the total duration represented by the simulation and calculates a value X^2 for the goodness-of-fit between the simulated data and the real FRAP data. Total simulation duration is selected as the one that produces the minimal X^2. Experiments were performed in biological replicas (N=3)**.**

imFCS analysis. imFCS measurements were performed as described earlier.[7,8] A home-built widefield setup with total internal reflection fluorescence (TIRF) illumination was used for imFCS analysis. The experimental setup was equipped with a 488 nm diode laser (100 mW, Obis, Coherent, USA). The excitation light passes through an acousto-optical tunable filter (AA Opto-Electronic, Orsay, France) and a telescope consisting of two achromatic lenses (Thorlabs, USA) with f = -40 mm and 750 mm. A third achromatic lens (f = 400 mm, Thorlabs) directed the excitation light to the TIRF mirror and had its focus on the back focal plane of the objective. The TIRF mirror was placed on a motorized translation stage (25 mm, #MTS25/M-Z8, Thorlabs) controlled by a motion controller (K-Cube Brushed DC Servo Motor Controller, #KDC101, Thorlabs) to switch between widefield and TIRF illumination. The light entered an Eclipse Ti microscope (Nikon, Japan) was reflected by a dichroic mirror (TIRF-Quad filter set 405/488/561/640 consisting of a QuadLine Laser Clean-up ZET405/488/561/640x, QuadLine dichroic zt405/488/561/640rpc, QuadLine rejection band ZET405/488/561/640 TIRF, all AHF

Analysentechnik AG, Tübingen, Germany), and was directed onto the sample by an oil-immersion TIRF objective (UapoN 100xOTIRF, 1.49 Oil, Olympus, Japan). A nosepiece stage (IX2-NPS, Olympus) was used for z-plane adjustment and drift minimization. Emission light was collected by the same objective and passed the dichroic mirror. In the detection path a TwinCam (Acal Bfi, Germany) with a BrightLine HC 525/45 bandpass filter (AHF Analysentechnik AG) was implemented, and the signal was detected by a scientific complementary metal-oxide semiconductor (sCMOS) camera (Zyla 4.2, Andor, Belfast, UK). Data were collected using the open-source software µManager.[9] For data acquisition the following settings were applied: 24 W/cm^2 laser intensity, a bit depth of 16 bit, pixel readout rate of 540 MHz, frame time 4 ms, 4x4 binning, and 4,000 frames per film. For each film, a 40x25 pixel (or 40x20 pixel) region of interest (ROI) was chosen and the measurement was performed with TIRF illumination to observe membrane diffusion of Y_2R. In total 36 untreated cells, 24 cells with *tris*NTA$^{PEG12-B}$ immobilized receptors, and 26 cells with anti-His$_6$ antibody immobilized receptors were measured. Each condition contains data from at least three independent measurement days (N=3).

imFCS data analysis. Analysis of imFCS films was performed using the imFCS plugin (version 1.52)[10] for Fiji[4]. The following correlation settings were chosen: emission wavelength = 515 nm, NA = 1.49, correlator scheme P = 16 and Q = 8, lateral PSF = 0.8, binning = 1, pixel size = 5.75 µm, magnification = 25 for 4x4 binning, and linear segment bleach correction with linear segments of 500 frames. Diffusion coefficients were obtained for each pixel by fitting the correlation curves according to the literature.[10] To compare the overall diffusion coefficients with those of the patterned regions, ROIs were placed around patterned regions and analyzed separately. For further analysis, the pixelwise diffusion coefficients for all measurements were imported into OriginPro 2019 (OriginLab Corporation, Northampton, USA). For box plots of diffusion coefficients, median diffusion coefficients were determined for each cell. Mean diffusion coefficients per condition were obtained by averaging over the median diffusion coefficients per measurement and calculating the standard error of the mean. Two-sample t-tests ($\alpha = 0.05$) were

applied to compare the diffusion coefficients for the different conditions. All datasets were tested for normality using the Kolmogorov-Smirnov test ($\alpha = 0.05$). Significance was assigned as follows: $p > 0.05$ no significant difference between populations (n.s.), $p < 0.05$ significant difference (*), $p < 0.01$ significant difference (**), and $p < 0.001$ significant difference (***). Two-dimensional maps of diffusion coefficients were generated also in OriginPro. Diffusion coefficients were color-coded from light yellow to dark red in the range of 0 to 0.5 µm²/s. Pixels that yielded correlation curves with diffusion coefficients higher than 0.5 µm²/s are presented in black. Pixels that yielded correlation curves which could not be fitted by the imFCS plugin in Fiji are shown in light grey. To generate frequency distribution plots, diffusion coefficients were log-transformed and binned in the interval between -5.3 and 1.0 with a bin size of 0.1 for each cell. Logarithmic diffusion coefficients were re-transformed, frequency counts were averaged over all cells per condition, and normalized. Frequency counts were plotted logarithmically against diffusion coefficients. Errors bars represent standard errors of the mean.

Arr3 recruitment upon receptor confinement. Microstructured surfaces were functionalized with biotin-BSA and SA or SA and anti-His_6 antibody as described before. For transient co-transfection with $Arr3^{mCherry}$, cells were sub-cultured the day before and then transfected with the $Arr3^{mCherry}$ plasmid using the TurboFect™ transfection reagent (Thermo Fisher Scientific), according to the manufacturer's instructions and induced with Tetracycline (0.1 µg/ml) 18 h before microscopy. Cells co-expressing His_6-Y_2R^{mGFP} and Arr3 were seeded onto the microstructured matrices and visualized by total internal reflection fluorescence (TIRF) microscopy in LCIS at 37 °C after 3 to 4 h to ensure a homogeneous cell membrane adhesion, which is a prerequisite for quantitative TIRF microscopy. For antibody experiments, cells grown on pre-structured matrices were incubated with pNPY (10 nM, Tocris) in LCIS for 30 min at 37 °C. For *tris*$NTA^{PEG12-B}$ experiments, cells grown on SA-matrices were subsequently incubated with nickel-loaded *tris*$NTA^{PEG12-B}$ (100 nM final) and pNPY (10 nM final) in LCIS for 30 min at 37 °C. For reversibility,

cells were incubated with histidine (5 mM) in LCIS for 30 min. Experiments were performed in biological replicas (N=2).

Arr3 imaging by TIRF microscopy. The detection system was set up on an epi-fluorescence microscope (Nikon Eclipse Ti2). For selective fluorescence excitation of mGFP and mCherry, a multi-laser engine (Toptica Photonics, Munich, Germany) was used at 488 and 561 nm, respectively. The samples were illuminated in total internal reflection (TIR) configuration (Nikon Ti-LAPP) using a 60x oil immersion objective (NA = 1.49, APON 60XO TIRF). After appropriate filtering using standard filter sets, the fluorescence was imaged onto a sCMOS camera (Zyla 4.2, Andor, Northern Ireland). The samples were mounted on an x-y-stage (CMR-STG-MHIX2-motorized table, Märzhäuser, Germany), and scanning was supported by a laser-guided automated Perfect Focus System (Nikon PFS).

Contrast quantification and statistical analyses. Contrast analysis was performed as described previously.[2,11,12] Initial imaging recording was supported by the Nikon NIS Elements software. Images were exported as TIFF frames and fluorescence contrast analysis was performed using the Spotty framework.[13] The fluorescence contrast <c> was calculated as $\langle c \rangle = (F^+ - F^-)/(F^+ - F_{bg})$, where F^+ denotes the intensity of the inner pixels of the pattern. F^- shows the intensity of the surrounding pixels of the micropattern, and F_{bg} the intensity of the global background. Data are expressed as the means ± SEM. Comparisons of more than two different groups were performed using one-way ANOVA, which was followed by Tukey’s multiple comparisons test in GraphPad Prism software (version 9.1.2).

SUPPLEMENTARY TEXT

Supplementary Text 1

NPY ligand plays an important role in the nervous, immune, and endocrine systems[14-16] and can affect the proliferation, apoptosis, differentiation, and migration of different cell types[17]. NPY is a potent angiogenic factor in vivo and the NPY-induced angiogenic response is mediated by the Y_2 receptor. In knock-out mice lacking the Y_2 receptor, skin wound repair was significantly delayed. Thus, NPY may play an important role in regulation of angiogenesis and angiogenesis-dependent physiological and pathological processes[18].

Further, NPY has recently been found to play a role in the progression of diverse types of cancer and diseases such as brain cancer, bone cancer, breast cancer and osteoporosis[19,20]. A rapid increase in bone mass was observed in adult mice after central Y_2R deletion suggesting that Y_2R may represent a promising new target for the prevention and treatment of osteoporosis. Recently, NPY or analogous small peptide agonists were tested as potential new strategies for the diagnosis or treatment of breast cancer or osteoporosis. However, these applications remained primarily in the research phase of animal testing[20]. In addition, an antinociceptive effect of spinally administrated NPY was recently observed in a cancer-induced bone pain model, mediated through both Y_1R and Y_2R, suggesting also that NPY might be a promising target for the development of future treatments for cancer-induced bone pain[21].

Supplementary Text 2

To corroborate the specificity of the interaction, clustering was evaluated in cells expressing Y_2Rs without the His_6-tag (Y_2R^{mEGFP}) cultured on SA-matrices. Cells showed no receptor clustering upon addition of *tris*$NTA^{PEG12-B}$ (Figure S2), demonstrating the specificity of the His_6-tag/*tris*NTA interaction.

With the aim to compare the nanotool-induced clustering with approaches using patterned antibodies, we utilized an anti-His_6 antibody. Ten minutes after receptor clustering by the multivalent nanotool, the Y_2R enrichment resulted in an integrated receptor density equivalent to that of cells cultured on matrices functionalized with anti-His_6 antibodies. However, a 10-fold higher antibody concentration (1 µM final) was required compared to the multivalent nanotool, demonstrating its efficacy in capturing His_6-tagged Y_2 receptors (Figure S3). In addition, the nanotool-induced 3 µm clusters presented a 9-fold increase in integrated density compared to 1 µm arrays, consistent with the increase in pattern area.

SUPPORTING REFERENCES

1 Gatterdam, K., Joest, E. F., Gatterdam, V. & Tampé, R. The scaffold design of trivalent chelator heads dictates affinity and stability for labeling His-tagged proteins in vitro and in cells. *Angew Chem Int Ed Engl* **57**, 12395-12399, doi:10.1002/anie.201802746 (2018).

2 Lanzerstorfer, P., Müller, U., Gordiyenko, K., Weghuber, J. & Niemeyer, C. M. Highly modular protein micropatterning sheds light on the role of clathrin-mediated endocytosis for the quantitative analysis of protein-protein interactions in live cells. *Biomolecules* **10**, 540, doi:10.3390/biom10040540 (2020).

3 Sánchez, M. F., Els-Heindl, S., Beck-Sickinger, A. G., Wieneke, R. & Tampé, R. Photoinduced receptor confinement drives ligand-independent GPCR signaling. *Science* **371**, eabb7657, doi:10.1126/science.abb7657 (2021).

4 Schindelin, J. *et al.* Fiji: an open-source platform for biological-image analysis. *Nat Methods* **9**, 676-682, doi:10.1038/nmeth.2019 (2012).

5 Schneider, C. A., Rasband, W. S. & Eliceiri, K. W. NIH Image to ImageJ: 25 years of image analysis. *Nat Methods* **9**, 671-675, doi:10.1038/nmeth.2089 (2012).

6 Blumenthal, D., Goldstien, L., Edidin, M. & Gheber, L. A. Universal approach to FRAP analysis of arbitrary bleaching patterns. *Sci Rep* **5**, 11655, doi:10.1038/srep11655 (2015).

7 Harwardt, M. I. E. *et al.* Membrane dynamics of resting and internalin B-bound MET receptor tyrosine kinase studied by single-molecule tracking. *FEBS Open Bio* **7**, 1422-1440, doi:10.1002/2211-5463.12285 (2017).

8 Harwardt, M. I. E., Dietz, M. S., Heilemann, M. & Wohland, T. SPT and imaging FCS provide complementary information on the dynamics of plasma membrane molecules. *Biophys J* **114**, 2432-2443, doi:10.1016/j.bpj.2018.03.013 (2018).

9 Edelstein, A., Amodaj, N., Hoover, K., Vale, R. & Stuurman, N. Computer control of microscopes using µManager. *Curr Protoc Mol Biol* **Chapter 14**, Unit14.20, doi:10.1002/0471142727.mb1420s92 (2010).

10 Sankaran, J., Shi, X., Ho, L. Y., Stelzer, E. H. & Wohland, T. ImFCS: a software for imaging FCS data analysis and visualization. *Opt Express* **18**, 25468-25481, doi:10.1364/oe.18.025468 (2010).

11 Schütz, G. J., Weghuber, J., Lanzerstorfer, P. & Sevcsik, E. Protein micropatterning assay: quantitative analysis of protein-protein interactions. *Methods Mol Biol* **1550**, 261-270, doi:10.1007/978-1-4939-6747-6_18 (2017).

12 Lanzerstorfer, P. *et al.* Quantification and kinetic analysis of Grb2-EGFR interaction on micro-patterned surfaces for the characterization of EGFR-modulating substances. *PLoS One* **9**, e92151, doi:10.1371/journal.pone.0092151 (2014).

13 Borgmann, D., Weghuber, J., Schaller, S., Jacak, J. & Winkler, S. M. in *Proceedings of the 24th European Modeling and Simulation Symposium.*

14 Chen, W. C. *et al.* Neuropeptide Y Is an Immunomodulatory Factor: Direct and Indirect. *Front Immunol* **11**, 580378, doi:10.3389/fimmu.2020.580378 (2020).

15 Yulyaningsih, E. *et al.* Pancreatic polypeptide controls energy homeostasis via Npy6r signaling in the suprachiasmatic nucleus in mice. *Cell Metab* **19**, 58-72, doi:10.1016/j.cmet.2013.11.019 (2014).

16 Eva, C., Oberto, A., Longo, A., Palanza, P. & Bertocchi, I. Sex differences in behavioral and metabolic effects of gene inactivation: The neuropeptide Y and Y receptors in the brain. *Neurosci Biobehav Rev* **119**, 333-347, doi:10.1016/j.neubiorev.2020.09.020 (2020).

17 Wu, J. Q., Jiang, N. & Yu, B. Mechanisms of action of neuropeptide Y on stem cells and its potential applications in orthopaedic disorders. *World J Stem Cells* **12**, 986-1000, doi:10.4252/wjsc.v12.i9.986 (2020).

18 Ekstrand, A. J. *et al.* Deletion of neuropeptide Y (NPY) 2 receptor in mice results in blockage of NPY-induced angiogenesis and delayed wound healing. *Proc Natl Acad Sci U S A* **100**, 6033-6038, doi:10.1073/pnas.1135965100 (2003).

19 Lin, S. T. *et al.* Update on the Role of Neuropeptide Y and Other Related Factors in Breast Cancer and Osteoporosis. *Front Endocrinol (Lausanne)* **12**, 705499, doi:10.3389/fendo.2021.705499 (2021).

20 Körner, M. & Reubi, J. C. Neuropeptide Y receptors in primary human brain tumors: overexpression in high-grade tumors. *J Neuropathol Exp Neurol* **67**, 741-749, doi:10.1097/NEN.0b013e318180e618 (2008).

21 Diaz-delCastillo, M. *et al.* Neuropeptide Y is Up-regulated and Induces Antinociception in Cancer-induced Bone Pain. *Neuroscience* **384**, 111-119, doi:10.1016/j.neuroscience.2018.05.025 (2018).

SUPPORTING FIGURES

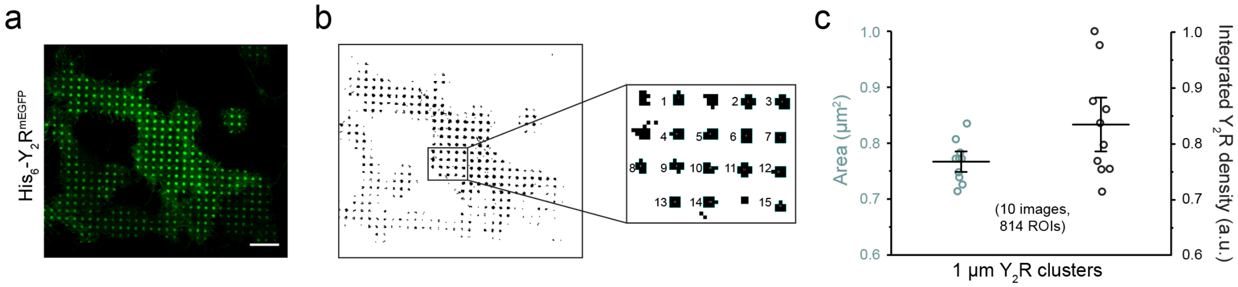

Figure S1. Receptor confinement with high reliability. (a) Representative confocal image of a cell patterned by *tris*NTA$^{PEG12\text{-}B}$. (b) Automatic cluster analysis performed by ImageJ requires a "binary", black and white, image. A threshold range is set to select the objects of interest apart from the background. All pixels in the image whose values lie under the threshold are converted to white and all pixels with values above the threshold are converted to black. Further selection of the clusters according to area and roundness enable a large-scale analysis. (c) Change in cluster area and integrated density of the receptor within different 96-well plates, different months and cell stocks reflected a reliable and reproducible approach. The average area in the clustered regions (0.77 ± 0.03 µm^2) and integrated density of ten images in five different experiments (841x ROIs of 1 µm in total) is shown. Scale bar: 10 µm.

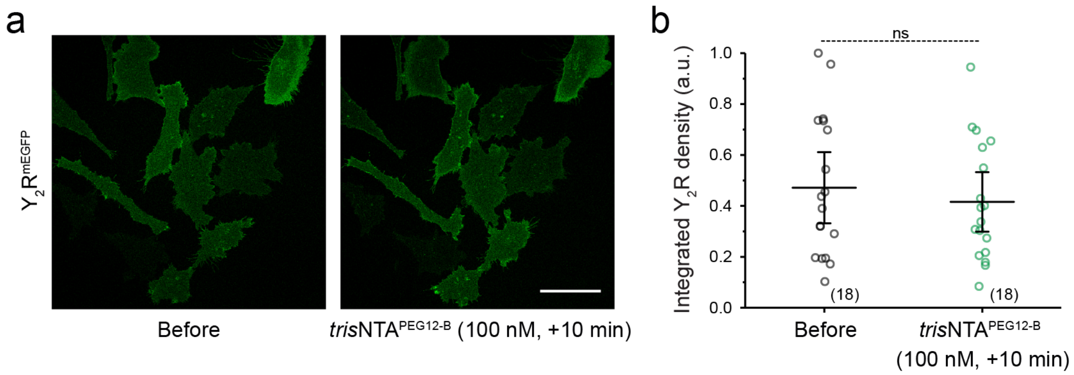

Figure S2. Y_2 receptors lacking a His_6-tag do not cluster in confined areas. (a) Representative confocal images of cells expressing Y_2 receptors without N-terminal His_6-tag over SA-pre-structured matrices before and after addition of the nanotool. Within the timeframe of imaging, there was neither a pattern formation nor a change in the integrated receptor density. (b) Quantification of the integrated Y_2R density before and after addition of *tris*NTA$^{PEG12-B}$. The mean ± SD (18 cells) is shown. **$p \leq 0.01$ for Tukey test. Scale bar: 50 µm.

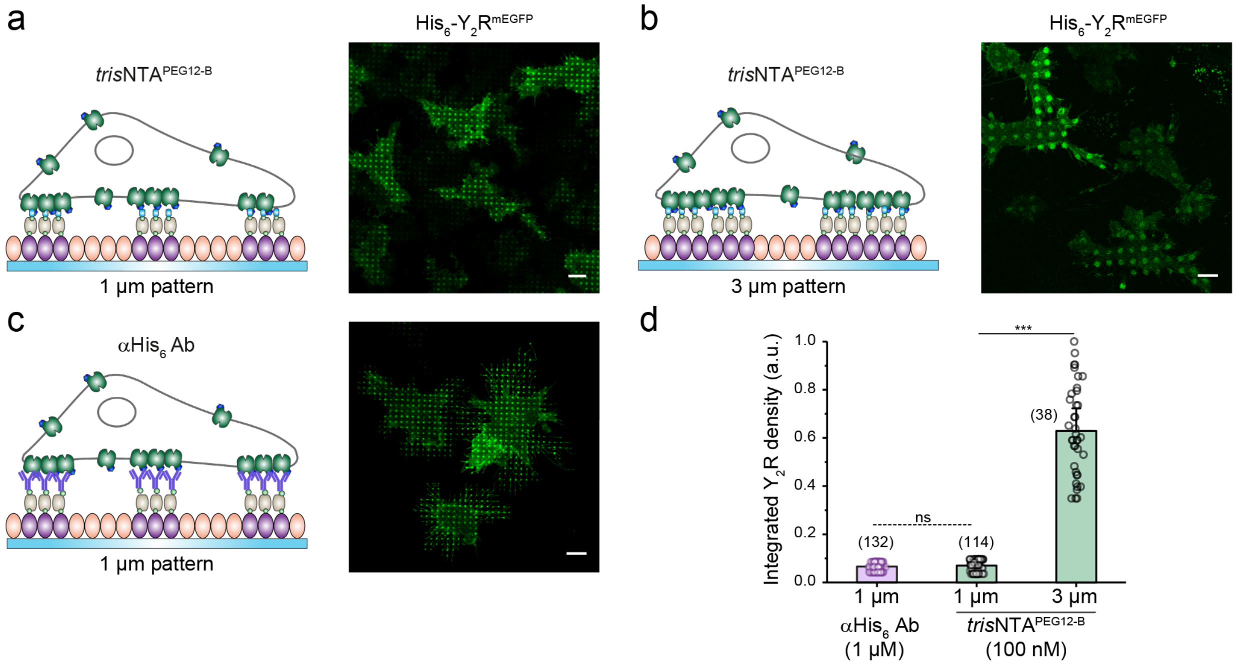

Figure S3. Receptor density correlates with the area of the pre-structured regions. (a, b) BSA-pre-structured matrices, 1 µm (a) or 3 µm (b), were stepwise functionalized with biotin-BSA and SA. Y_2R-expressing HeLa cells were allowed to adhere to the functionalized matrix for 3 h and immediately imaged by CLSM in live-cell imaging solution (LCIS) at 37 °C. Incubation with *tris*NTA$^{PEG12-B}$ (100 nM final) led to *in situ* receptor assembly. (c) 1 µm BSA-pre-structured matrices were stepwise functionalized with biotin-BSA, SA, and a biotinylated anti-His_6 antibody. Y_2R-expressing cells were allowed to adhere to the functionalized matrix for 3 h and immediately imaged by CLSM in LCIS at 37 °C. (d) Quantification of the receptor-integrated density. *In situ* receptor confinement by *tris*NTA$^{PEG12-B}$ resulted in a receptor density that is comparable to cells in contact with pre-structured antibodies. For the 3 µm patterns, receptor density correlated with pattern area. The mean ± SD (38 to 132x 1 µm ROIs) is shown. ***p≤ 0.001 for Tukey test. Scale bars: 10 µm.

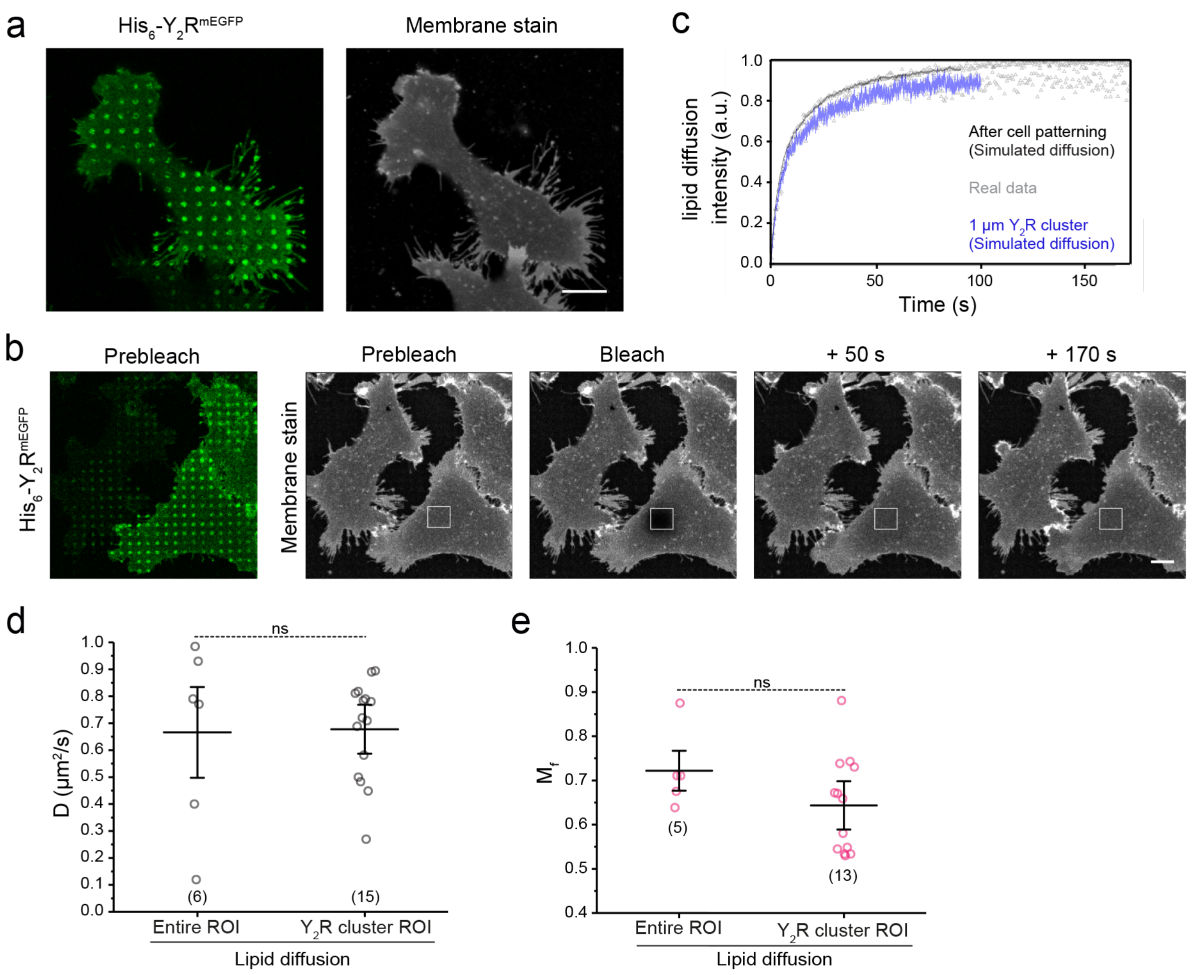

Figure S4. Lipid localization and dynamics after receptor confinement. (a) Confocal microscopy images of the live-cell plasma membrane staining, which was performed 15 min after Y_2R assembly in living cells. 5 µg/ml CellMask staining solution was incubated for 5 min at 37 °C and washed with LCIS before visualization. Lipid distribution is not affected by receptor confinement as shown by the homogeneous staining of the membrane. (b, c) FRAP recovery curve (b) and time-lapse (c) for the lipid dye demonstrated a rapid recovery for the lipids. Diffusion was measured in the entire rectangular ROI or at the Y_2R cluster spots (region selected based on the receptor channel image). An image of the receptor channel confirmed the presence of the pattern. (d) The analysis did not show any differences in lipid diffusion coefficients for the entire rectangular ROI or at the 1 µm clustered regions ($D_{\text{entire ROI}}$ = 0.66 ± 0.10 µm^2/s and D_{spots} = 0.67 ± 0.17 µm^2/s). The mean ± SD (6 cells, 15x 1 µm ROIs) is shown. **p≤ 0.01 for Tukey test. (e) Quantification of the mobile fraction (M_f) for FRAP measurements of the lipid dye reflected no significant difference. The mean ± SD (5 cells, 14x 1 µm ROIs) is shown. **p≤ 0.01 for Tukey test. Scale bars: 10 µm.

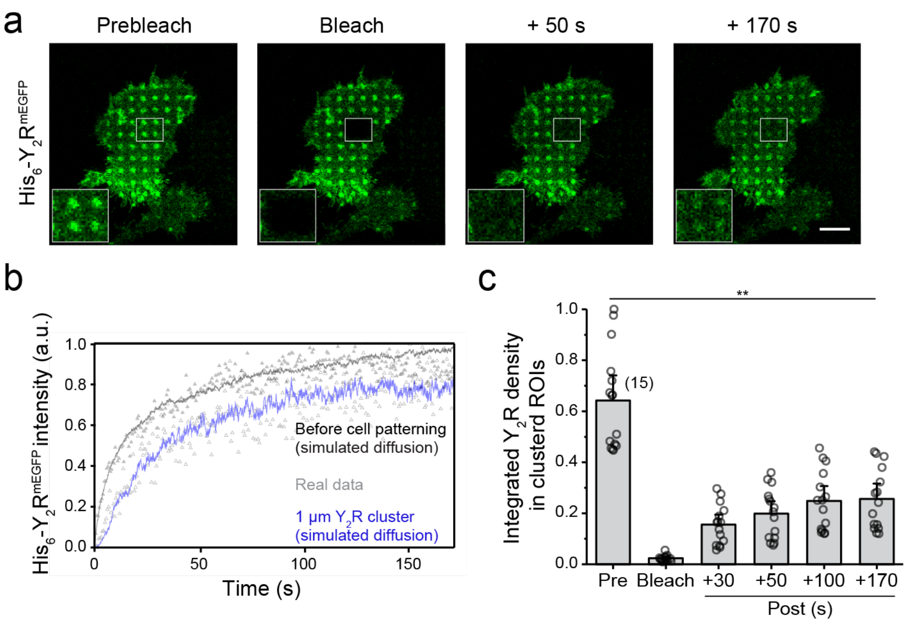

Figure S5. Dynamic receptor exchange in confined clusters. (a) Representative confocal images of FRAP measurements for Y_2R-expressing cells on SA-pre-structured matrices 10 min after addition of the nanotool. (b) FRAP recovery curves reflecting the entire bleached area or an analysis performed only in the clustered 1 µm regions. The analysis is based on a simulation approach which fits a computer-simulated recovery to actual recovery data of a FRAP series and determines the diffusion coefficient regardless of bleaching geometry. (c) Quantification of the receptor density in the confined regions showed 50% recovery indicating a high exchange rate. The mean ± SD (6 cells, 15x 1 µm ROIs analyzed) is shown. **$p \leq 0.01$ for Tukey test. Scale bar: 10 µm.

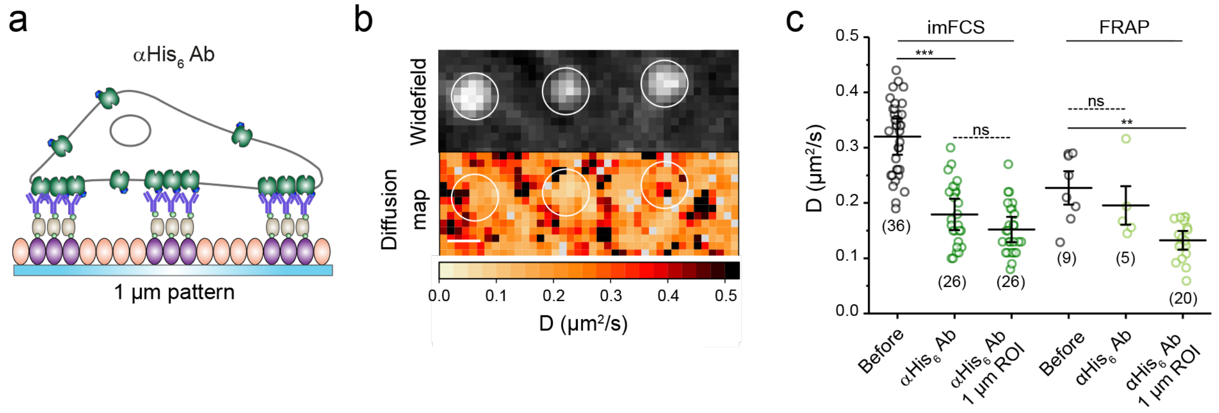

Figure S6. Receptor mobility on antibody structured matrices. (a) Scheme representing the experimental set-up. (b) Representative widefield image (left) of a ROI at the plasma membrane of a living cell over a pre-structured matrices with an anti-His_6 antibody analyzed by imFCS and the derived two-dimensional diffusion map (right). (c) Lateral diffusion of the receptor analyzed by FRAP and imFCS. Both techniques demonstrated a decrease in D at the plasma membrane (D_{before}= 0.32 ± 0.06 µm^2/s and 0.25 ± 0.08 µm^2/s; $D_{\text{anti-His6 Ab}}$ = 0.18 ± 0.06 and 0.19 ± 0.06 µm^2/s for imFCS and FRAP, respectively), concurring with the values obtained for the measurements upon addition of the nanotool. Analysis of clustered regions (1 µm) within the selected ROIs led to a further decrease in the diffusion coefficient (D_{spots}= 0.15 ± 0.05 µm^2/s and 0.13 ± 0.03 µm^2/s for imFCS and FRAP, respectively). For imFCS measurements, two-sample t-tests (α = 0.05) were applied to compare the diffusion coefficients for the different conditions (***$p \leq 0.001$). The mean ± SD is shown. 36 and 26 cells for the conditions before and after addition of anti-His_6 antibody were analyzed. For FRAP, the mean ± SD is shown. Here, 9 cells before, 5 cells after addition of anti-His_6 antibody, 20x 1 µm ROIs were examined. **$p \leq 0.01$ for Tukey test. Scale bar: 1 µm.

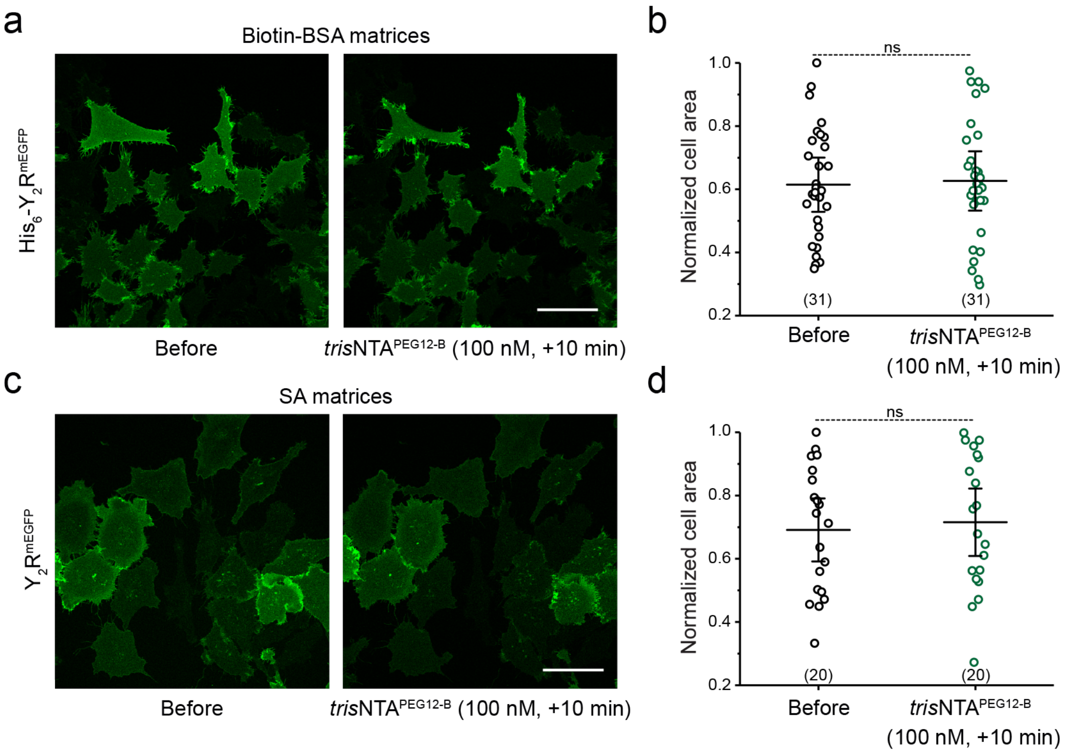

Figure S7. Changes in cell motility are exclusively triggered upon receptor clustering. (a, b) Confocal images of cells expressing His_6-tagged Y_2R on matrices which do not contain SA but biotin-BSA only. Addition of the *tris*$NTA^{PEG12\text{-}B}$ nanotool confirmed no effect on cell spreading and motility as shown in the quantification of the cell area (b). The mean ± SD (31 cells) is shown. **p≤ 0.01 for Tukey test. (c) Confocal images of cells expressing Y_2 receptors lacking the His_6-tag on SA-matrices do not present significant changes in cells spreading upon addition of the nanotool. (d) Quantification of the cell area before and after addition of *tris*$NTA^{PEG12\text{-}B}$ (100 nM). Values for cell area were normalized with respect to the highest value. The mean ± SD (20 cells) is shown. **p≤ 0.01 for Tukey test. Scale bar: 50 µm.

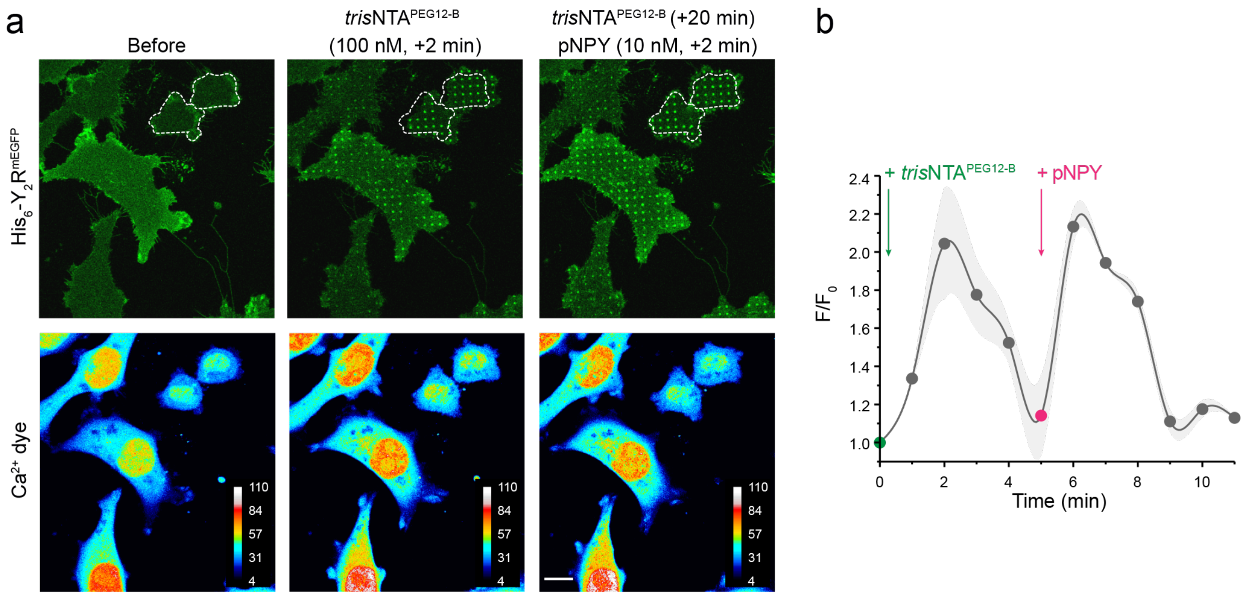

Figure S8. Ligand-free receptor confinement provokes calcium signaling. (a) Representative confocal fluorescence images of the Y_2R (upper panel) and color-coded images of the Ca^{2+} dye (lower panel). Y_2R-expressing cells on SA-pre-structured matrices were incubated with BioTracker 609 Red Ca^{2+} AM dye (3 µM) for 30 min. After rinsing, cells were immediately imaged by CLSM in LCIS at 37 °C. Addition of *tris*NTA$^{PEG12\text{-}B}$ showed a 2-fold increase in cytosolic calcium. Scale bar: 10 µm. (b) Analysis of the mean gray value for Ca^{2+} signal before (F_0) and upon addition of *tris*NTA$^{PEG12\text{-}B}$ (F) *versus* time. Time-lapse images were recorded with 45 s interval before and after addition of *tris*NTA$^{PEG12\text{-}B}$ (100 nM), and subsequent addition of pNPY (10 nM) after 5 min of nanotool addition (5 slices z-stack per time-point). ROIs covering the complete cell area were considered. The mean ± SD (10 cells) is shown.

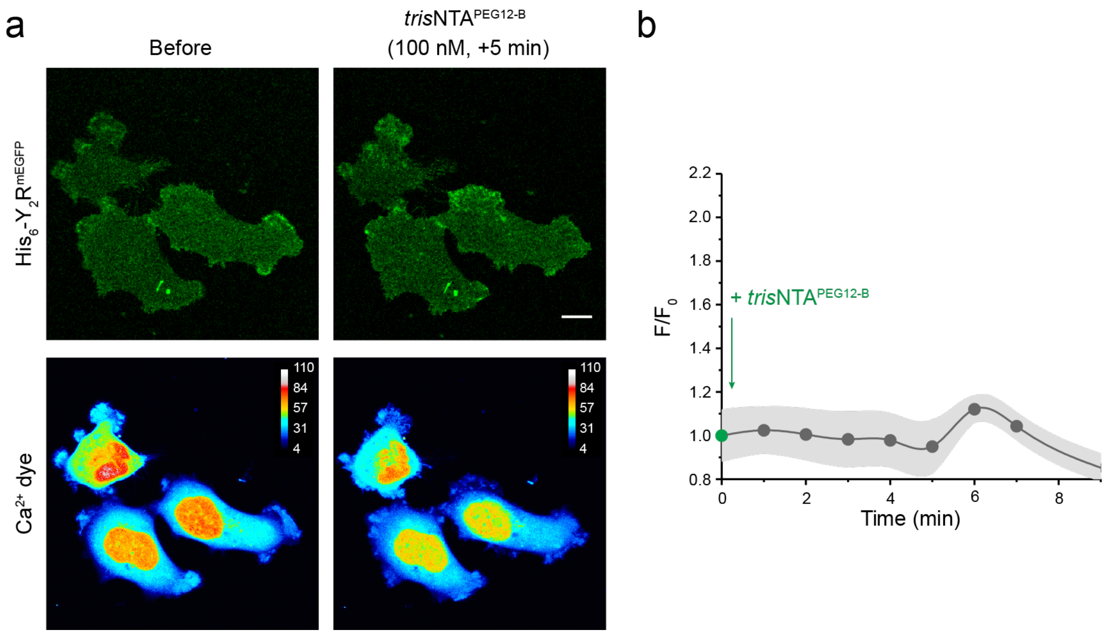

Figure S9. Calcium signaling is a specific response upon clustering. (a) Representative fluorescence images of the Y_2R (upper panel) and color-coded images of the Ca^{2+} dye (lower panel). Y_2R-expressing cells over pre-structured matrices in the absence of streptavidin were incubated with BioTracker 609 Red Ca^{2+} AM dye (3 µM) for 30 min. After rinsing, cells were immediately imaged by CLSM in LCIS at 37 °C. Addition of *tris*NTA$^{PEG12-B}$ showed neither clustering nor change in cytosolic calcium. Scale bar: 10 µm. (b) Analysis of the mean gray value for Ca^{2+} signal before (F_0) and upon (F) addition of *tris*NTA$^{PEG12-B}$ *versus* time. Time-lapse images were recorded with 45 s interval before and after addition of *tris*NTA$^{PEG12-B}$ (100 nM) (5 slices z-stack per time-point). ROIs covering the complete cell area were considered. The mean ± SD (10 cells) is shown.

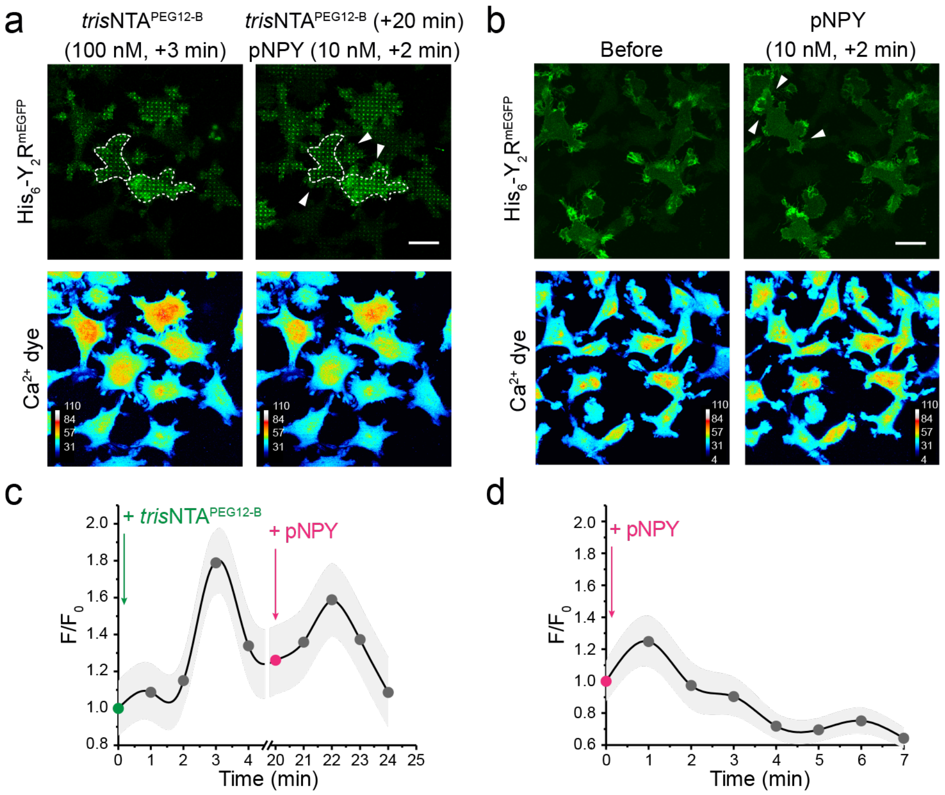

Figure S10. Receptor clustering potentiates calcium signaling. (a, b) Representative fluorescence images of the Y_2R (upper panel) and color-coded images of the Ca^{2+} dye (lower panel). Y_2R-expressing HeLa cells were allowed to adhere to pre-structured SA-matrices for 3 h. Before visualization, cells were incubated with BioTracker 609 Red Ca^{2+} AM dye (3 µM) for 30 min. After rinsing, cells were visualized by CLSM in LCIS at 37 °C and imaged before and after addition of only pNPY or before and after *tris*NTA$^{PEG12\text{-}B}$ and subsequent addition of pNPY. Scale bar: 20 µm. (c, d) Analysis of the mean gray value for Ca^{2+} signal before (F_0) and upon (F) addition of *tris*NTA$^{PEG12\text{-}B}$/pNPY versus time. Time-lapse images were recorded with 45 s interval before and after addition of *tris*NTA$^{PEG12\text{-}B}$/pNPY (5 slices z-stack per time-point). ROIs covering the complete cell area were considered. The mean ± SD (10 cells for each condition) is shown.

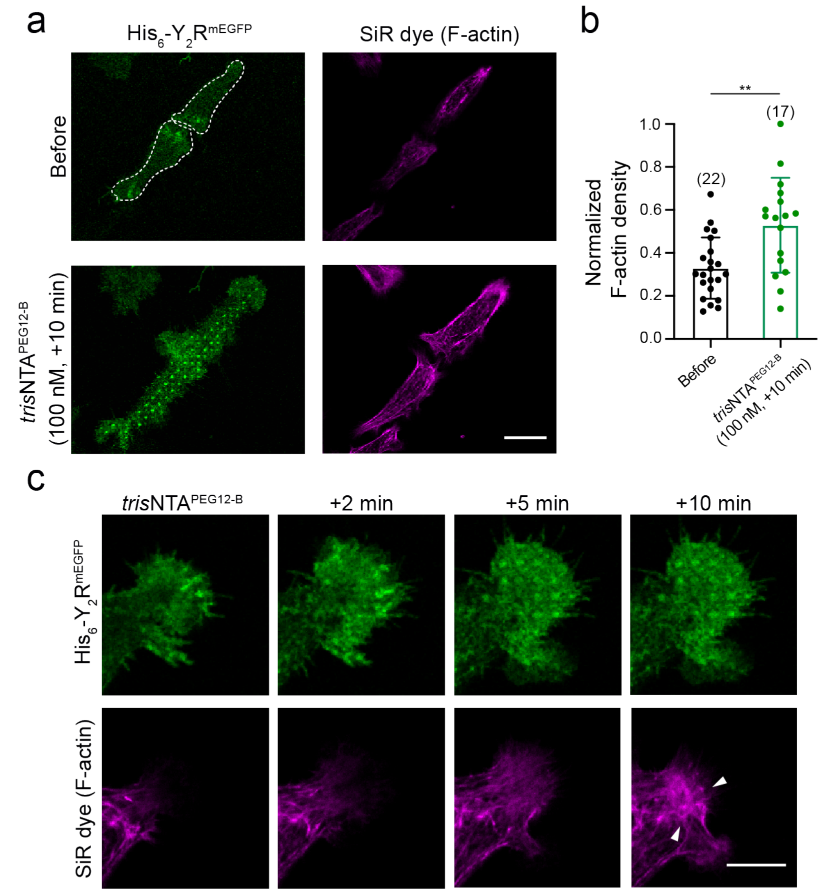

Figure S11. Receptor clustering induced an increase actin density. (a) Representative fluorescence images of the Y_2R (left panel) and color-coded images of the SiR-actin dye (right panel). Y_2R-expressing HeLa were allowed to adhere to pre-structured SA-matrices for 3 h. Before visualization, cells were incubated with SiR-actin dye (100 nM) for 15 min. After rinsing, cells were visualized by CLSM in LCIS at 37 °C and imaged before and after addition of *tris*NTA$^{PEG12\text{-}B}$. Scale bar: 10 µm. (b) Analysis of the F-actin integrated density signal before and upon addition of *tris*NTA$^{PEG12\text{-}B}$. The mean ± SD (22 cells before and 17 cells after) is shown. (c) Time-lapse images of a cell tip immediately after addition of the nanotool show increased enrichment of actin with time. Scale bar: 10 µm.

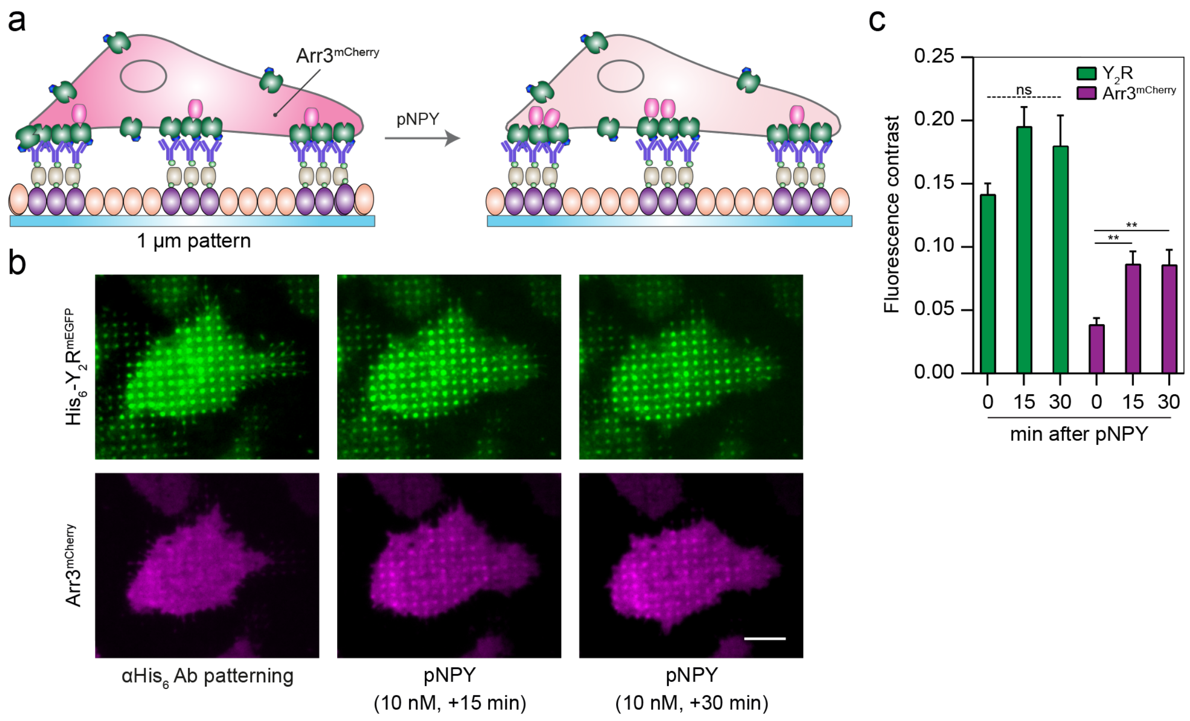

Figure S12. Arrestin-3 recruitment on antibody-confined regions. (a) Schematic representation of the experimental set-up. Cells co-expressing Y_2R and Arr3 were allowed to adhere to anti-His_6 antibody pre-structured matrices for 3 h and visualized by total internal reflection fluorescence (TIRF) microscopy in LCIS at 37 °C. (b) Representative TIRF images of cells before and upon addition of pNPY (10 nM) in LCIS for 30 min at 37 °C. Scale bar: 10 µm. (c) Quantification of the fluorescence contrast in the Y_2R-patterned regions showed no significant change in receptor intensity yet a recruitment of Arr3 upon addition of pNPY (2-fold). Data are expressed as the means ± SEM (30 cells for each condition were analyzed). Tukey's multiple comparison test was applied (**$p \leq 0.01$).

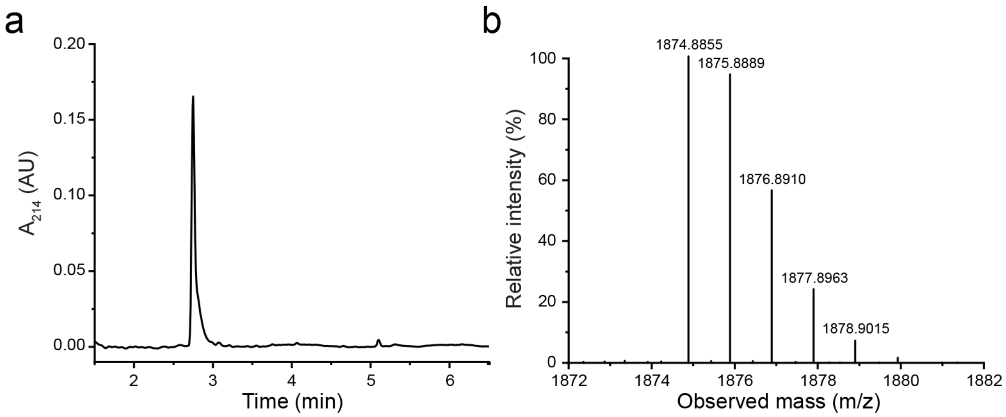

Figure S13. Multivalent nanotool *tris*NTA$^{PEG12\text{-}B}$ analyzed by LC-MS. (a) *tris*NTA$^{PEG12\text{-}B}$ chromatogram reflecting the purity of the synthesized nanotool. (b) LC-MS of *tris*NTA$^{PEG12\text{-}B}$, yielding the experimental mass ($M_{exp.}$) of 1874.85 Da ($M_{theor.}$ = 1873.90 Da).

SUPPORTING VIDEOS

Video 1

Time-lapse after photobleaching of a region of interest containing Y_2R clusters induced by the nanotool. A fast recovery is observed within the first minutes indicating a high dynamic behavior for these clusters.

Video 2

Time-lapse after photobleaching of a region of interest containing Y_2R clusters induced by an anti-His_6 tag antibody immobilized on the pre-structured matrices. There is no recovery of the clusters indicating a high degree of immobilization.

Video 3

Time-lapse immediately after the addition of the nanotool to Y_2R expressing cells over SA pre-structured matrices show fast enrichment of the receptors in real-time.
